# Deubiquitylase USP52 Promotes Bladder Cancer Progression by Modulating Ferroptosis through Stabilizing SLC7A11/xCT

**DOI:** 10.1002/advs.202403995

**Published:** 2024-10-11

**Authors:** Jianmin Liu, Yongwen Luo, Siming Chen, Gang Wang, Wan Jin, Wenyu Jiang, Mingxing Li, Yejinpeng Wang, Jingtian Yu, Houyi Wei, Renjie Zhang, Fenfang Zhou, Lingao Ju, Yi Zhang, Yu Xiao, Kaiyu Qian, Xinghuan Wang

**Affiliations:** ^1^ Department of Urology Zhongnan Hospital of Wuhan University Wuhan 430071 China; ^2^ Department of Biological Repositories Human Genetic Resources Preservation Center of Hubei Province Zhongnan Hospital of Wuhan University Wuhan 430071 China; ^3^ Hubei Key Laboratory of Urological Diseases Zhongnan Hospital of Wuhan University Wuhan 430071 China; ^4^ Euler Technology ZGC Life Sciences Park Beijing 102206 China; ^5^ Department of Radiology Zhongnan Hospital of Wuhan University Wuhan 430071 China; ^6^ Wuhan Research Center for Infectious Diseases and Cancer Chinese Academy of Medical Sciences Wuhan 430071 China; ^7^ Medical Research Institute Frontier Science Center for Immunology and Metabolism Taikang Center for Life and Medical Sciences Wuhan University Wuhan 430071 China

**Keywords:** bladder cancer, deubiquitylases, ferroptosis, SLC7A11/xCT, USP52

## Abstract

Bladder cancer (BLCA) is a prevalent cancer with high case‐fatality rates and a substantial economic burden worldwide. Understanding its molecular underpinnings to guide clinical management is crucial. Ferroptosis, a recently described non‐apoptotic form of cell death, is initiated by the lethal accumulation of iron‐dependent lipid peroxidation products. Despite growing interest, the roles and vulnerabilities determining ferroptosis sensitivity in BLCA remain unclear. Re‐analysis of single‐cell RNA data reveals a decrease in high‐ferroptosis cancer cells as BLCA advances. USP52/PAN2 is identified as a key regulator of ferroptosis in BLCA through an unbiased siRNA screen targeting 96 deubiquitylases (DUBs). Functionally, *USP52* depletion impedes glutathione (GSH) synthesis by promoting xCT protein degradation, increasing lipid peroxidation and ferroptosis susceptibility, thus suppressing BLCA progression. Mechanistically, USP52 interacts with xCT and enzymatically cleaves the K48‐conjugated ubiquitin chains at K4 and K12, enhancing its protein stability. Clinical BLCA samples demonstrate a positive correlation between USP52 and xCT expression, with high USP52 levels associated with aggressive disease progression and poor prognosis. In vivo, *USP52* depletion combined with ferroptosis triggers imidazole ketone Erastin (IKE) synergistically restrains BLCA progression by inducing ferroptosis. These findings elucidate the role of the USP52‐xCT axis in BLCA and highlight the therapeutic potential of targeting USP52 and ferroptosis inducers in BLCA.

## Graphical abstract

An siRNA screen targeting 96 deubiquitinases identifies USP52/PAN2 as crucial for xCT stability and ferroptosis in bladder cancer (BLCA). USP52 depletion impedes glutathione synthesis by promoting xCT degradation, increasing lipid peroxidation and ferroptosis, thereby suppressing BLCA progression. High USP52 levels correlate with aggressive disease and poor prognosis. In vivo, combining USP52 depletion with ferroptosis triggers IKE effectively restrains BLCA progression.

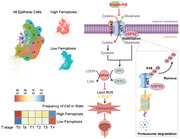

## 1 Introduction

Bladder cancer (BLCA) ranks as the 10th most common cancer, with an estimated 573 thousand new cases diagnosed and 212 thousand cancer deaths occurring worldwide in 2020.^[^ ^1^ ^]^ In clinical practice, multimodal therapeutic approaches, including surgery, systemic chemotherapy, and radiotherapy, are paramount for improving patient survival. However, even with early aggressive therapy, at least 50% of patients eventually experience recurrence and have an inferior prognosis.^[^ ^2^ ^]^ Therefore, more research efforts are required to explore the potential molecular underpinnings of BLCA tumorigenesis and progression and to further identify diagnostic and prognostic biomarkers to guide the clinical management of BLCA.

Ferroptosis, a newly defined iron‐dependent regulated cell death (RCD) triggered by excessive lethal build‐up of lipid peroxidation on cellular membranes,^[^ ^3^ ^]^ is morphologically, biochemically, and genetically distinct from other forms of RCD, such as autophagy, necroptosis, and apoptosis.^[^ ^4^ ^]^ Solute carrier family 7 membrane 11 (SLC7A11, also known as xCT), a 12‐pass transmembrane protein, is the catalytic subunit of the cystine/glutamate transporter system x_c_^−^ and functions to import cystine into cells via a 1:1 counter transport of glutamate.^[^ ^5^ ^]^ Once in cells, cystine is quickly converted to cysteine, which is subsequently utilized for the synthesis of glutathione (GSH). Glutathione peroxidase 4 (GPX4) uses GSH to enzymatically reduce lipid hydroperoxides to lipid alcohols to protect cells against membrane lipid peroxidation and thereby exert a suppressive effect on ferroptosis.^[^ ^6^ ^]^ A furry amount of evidence demonstrates that the induction of ferroptosis may play a significant role, either alone or in combination, in tumor suppression.^[^ ^7^ ^]^ Despite the dysregulation of ferroptosis in cancer cells is emerging as a promising target in cancer therapy,^[^ ^7^, ^8^ ^]^ the role and internal mechanism of ferroptosis in BLCA is still limited.

As a key regulator of ferroptosis, the diverse regulatory mechanisms of xCT in cancer have been largely established. In response to oxidative stress conditions, the proteasomal degradation of Kelch‐like ECH‐associated protein‐1 (KEAP1)‐mediated nuclear factor erythroid 2‐related factor 2 (NRF2) is inhibited, thereby stabilizing NRF2 protein translocation into the nucleus and inducing the transcription of xCT.^[^ ^9^ ^]^ Under basal conditions, p53^[^ ^10^ ^]^ and ATF3^[^ ^11^ ^]^ repress xCT transcription. In addition, activating transcription factor 4 (ATF4),^[^ ^12^ ^]^ BRCA1‐associated protein‐1 (BAP1)^[^ ^13^ ^]^ and the RNA‐binding protein RBSM1^[^ ^14^ ^]^ are involved in the transcriptional regulation of xCT. As such, the transcriptional control of xCT has been well studied, whereas the regulation of xCT at the posttranslational level remains relatively preliminary. For instance, OTUB1, an ovarian tumor family member deubiquitinase (DUB), and CD44 form a trimeric complex with xCT to deubiquitinate and stabilize the xCT protein, thereby increasing resistance to ferroptosis.^[^ ^15^ ^]^

DUBs, comprising ≈100 members in mammals, modulate target substrates by specifically releasing ubiquitin chains.^[^ ^16^ ^]^ Unsurprisingly, DUBs exhibit a strong correlation with the onset and progression of cancer, particularly in the context of BLCA. Recently, our group reveal that several DUBs are involved in BLCA tumorigenesis through diverse regulatory mechanisms.^[^ ^17^ ^]^ Ubiquitin‐specific peptidase 52/poly(A) specific ribonuclease subunit 2 (USP52/PAN2), a member of the ubiquitin‐specific peptidase (USP) class, contains an N‐terminal WD40 domain, a ubiquitin C‐terminal hydrolase (UCH) domain, and an exonuclease (EXO) domain at the C‐terminus.^[^ ^18^ ^]^ A recent study revealed that USP52 physically interacts with and deubiquitinates the histone chaperone anti‐silencing function 1A (ASF1A) to reduce the susceptibility of tumor cells to DNA damage and promote breast carcinogenesis.^[^ ^18b^ ^]^ In addition, USP52 deubiquitinates C‐terminal binding protein‐interacting protein (CtIP), thereby promoting DNA end resection and homologous recombination.^[^ ^19^ ^]^ Also, USP52 exerts a role in regulating the type I interferon signaling pathway during antiviral responses.^[^ ^20^ ^]^ These findings indicated that USP52 not only acts as a nuclease to improve mRNA stability but also acts as a bone fide DUB to stabilize substrate proteins in modulating a variety of biological processes. Interestingly, USP52 is low expressed as a tumor suppressor gene in lung cancer, but overexpressed as an oncogene in breast cancer.^[^ ^18^, ^21^ ^]^ However, its role in BLCA has not been characterized.

Here, our study showed an oncogene role of USP52 and a tumor suppressor role of ferroptosis in BLCA, with high USP52 levels associated with more aggressive disease progression and poorer prognosis. Besides, USP52 was systematically screened as a cardinal regulator of ferroptotic cell death. Depletion of USP52 sensitized tumor cells to ferroptosis by repressed xCT protein stabilization through cleaving the K48‐conjugated ubiquitin chains of xCT at K4 and K12, consequently suppressing BLCA progression. Combined USP52 depletion and ferroptosis induction hold tantalizing promise in the therapeutic application of BLCA.

## 2 Results

### 2.1 Systematic Screening of USP52 as a Potential Regulator of Ferroptosis in BLCA

**Figure 1:**
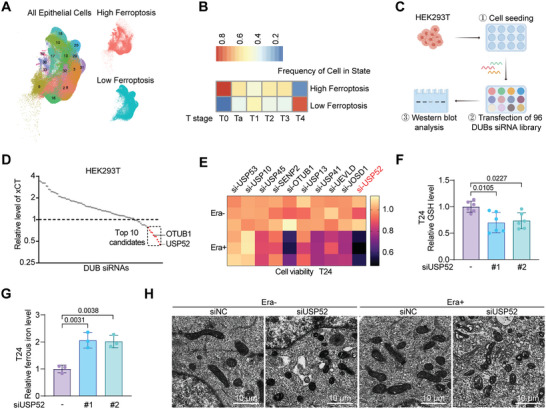
Systematic RNAi screening identified USP52 as a key regulator of ferroptosis and xCT translational activity in bladder cancer. A) UMAP projection of scRNA‐seq data from bladder tissues. All epithelial cells, cells in a high ferroptosis state, and cells in a low ferroptosis state are shown. B) The frequency of cells in high and low ferroptosis states across BLCA clinical T‐stages including T0 (indicated healthy organ donor), Ta, T1, T2, T3, and T4. C) Flow chart of 96 DUBs siRNAs screening. HEK293T cells were seeded and transfected with negative control siRNA or 25 nm DUBs siRNAs for 3 days in the 12‐well plates. The cells were collected for total protein extraction and subsequent Western blot analysis. This Figure was drawn by an online tool Figdraw (https://www.figdraw.com/static/index.html#/paint_index_v2). D) Statistic analysis of the relative xCT protein levels after transfection of DUBs siRNAs library via Western blotting with GAPDH as a normalization control. The intensity of each band was quantified by densitometry with ImageJ software. OTUB1, USP52, and 8 other DUBs were among the top 10 hits, the inhibition of which markedly suppressed xCT protein expression. E) T24 bladder cancer cells were transfected with 25 nm siRNAs targeting the top 10 candidate DUBs for 24 h, treated with or without 2 µm Erastin for 48 h, and finally subjected to an MTT assay. F) Control or *USP52*‐specific siRNAs were transfected into T24 cells for 48 h in the 6‐well plates. Then, seeded ≈5000 transfected cells per well on the 96‐well plates for 24 h, followed by an assessment of the relative GSH level with a microplate reader. G) Control or *USP52*‐specific siRNAs were transfected into T24 cells for 48 h in the 6‐well plates. Then, collected cells and assessed the relative ferrous iron level with a microplate reader. H) *USP52*‐silenced T24 cells were treated with or without 2 µm Erastin (Era) for 48 h, followed by collection and fixation, and finally subject to observation by transmission electron microscopy. The ultrastructure of the mitochondria was captured. Scale bars: 10 µm. The data are presented as the means ± SDs, with *n* = 3 (E, G) or 6 (F) independent repeats. Statistical significance was determined by one‐way ANOVA with Tukey's multiple comparisons (F, G).

To characterize BLCA evolution at the single‐cell (sc) level, our research group performed single‐cell RNA sequencing (scRNA‐seq) on bladder cancer tissues across all stages.^[^ ^22^ ^]^ To investigate whether ferroptosis is involved in the progression of BLCA, we scored ferroptosis‐related gene set expression levels in each individual single epithelial cell and categorized them into high or low ferroptosis group according to the expression levels of ferroptosis‐related genes (**Figure** **1**A). The prevalence of high‐ferroptosis cancer cells decreases as the BLCA clinical stage advances (Figure 1B). Overall, ferroptosis may exert a potential suppressive effect on BLCA. Thus, gaining an in‐depth understanding of ferroptosis mechanisms can offer crucial insights into targeting this process in BLCA.

DUBs have essential roles in maintaining the stability of the majority of proteins and have been linked to ferroptosis. Based on our data and previous cognition of the cardinal role of xCT in ferroptosis,^[^ ^23^ ^]^ we hypothesized that high xCT protein regulated by the deubiquitination modification might contribute to ferroptosis resistance in BLCA. Treatment of BLCA would benefit from the combination of ferroptosis induction and DUB‐specific interference. To this end, using alterations in xCT protein levels as a terminal effect, we performed a siRNA screen targeting 96 DUBs in HEK293T cells (Figure 1C). The efficiency of the DUB siRNA library used in our study was validated by the laboratories of Qing^[^ ^24^ ^]^ and Tao.^[^ ^25^ ^]^ Silencing each DUB resulted in an obvious change in xCT protein levels (Figure S1A, Supporting Information). Among these 96 DUBs, *USP52* knockdown resulted in the most dramatic decrease in xCT levels, with the reported DUB OTUB1, which is responsible for xCT deubiquitylation,^[^ ^15b^ ^]^ also ranking among the top 10 candidates (Figure 1D). Then, we examined the roles of these 10 candidates in cell viability with or without treatment with Erastin, a compound that blocks xCT‐mediated cystine transport to induce ferroptosis.^[^ ^26^ ^]^ Notably, T24 cells depleted of *USP52* exhibited heightened sensitivity to Erastin‐induced ferroptosis (Figure 1E), leading to the selection of USP52 as the final candidate.

To further explore the potential roles of USP52 in regulating ferroptosis, we generated transiently *USP52*‐silenced and USP52‐overexpressing bladder cancer cells with independent siRNAs and plasmids, respectively. The knockdown or overexpression efficiency was confirmed at the transcriptional and translational levels (Figure S1B–E, Supporting Information). GSH, synthesized from cysteine, serves as one of the most important antioxidants by scavenging reactive oxygen species (ROS), particularly lipid ROS, thereby regulating sensitivity to ferroptosis.^[^ ^5^ ^]^ As indicated in Figure 1F, the knockdown of *USP52* led to a decrease in the cellular GSH concentration. It was reported that iron accumulation is another main biochemical characteristic of ferroptosis.^[^ ^27^ ^]^ As shown in Figure 1G, the relative ferrous iron level was significantly upregulated after *USP52* depletion. Furthermore, transmission electron microscopy (TEM) analysis revealed shrunken mitochondria, a hallmark of ferroptosis, in *USP52*‐silenced cells (Figure 1H). Altogether, these results demonstrated that USP52 was identified as a potential regulator of ferroptosis in BLCA.

### 2.2 Depletion of *USP52* Sensitizes BLCA Cells to Ferroptosis

**Figure 2:**
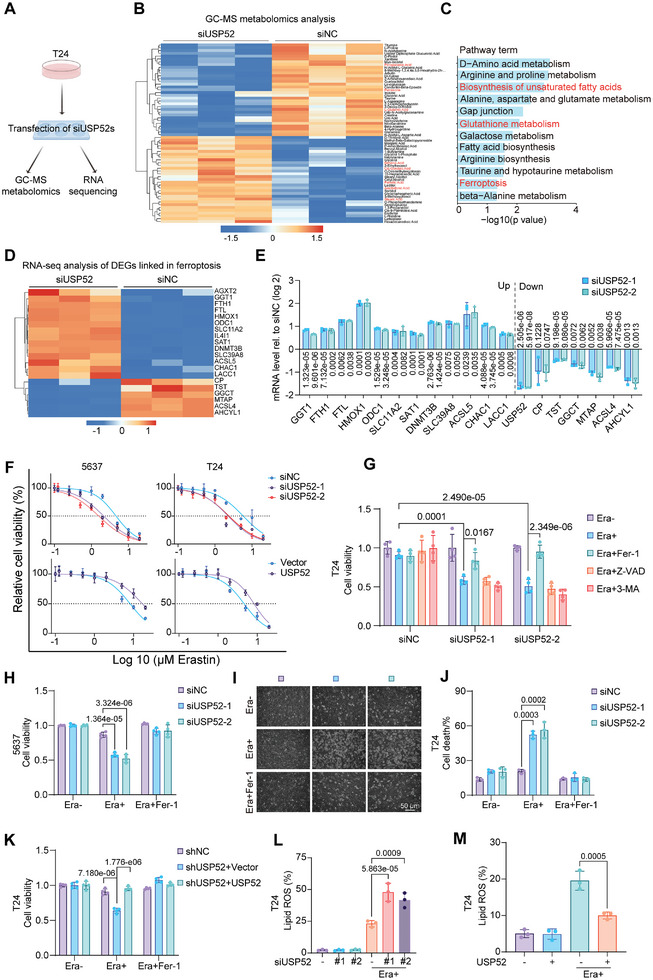
*USP52* depletion promotes ferroptosis in bladder cancer cells. A) Flow chart of the RNA‐seq and GC‐MS metabolomic analyses. We transfected T24 cells with *siNC* and *siUSP52s* in the 6‐well plates for 2 days. Then, a quarter of the cells were performed RNA‐seq, and the remaining three quarters were conducted for GC‐MS metabolomic analyses. This Figure was drawn by an online tool Figdraw (https://www.figdraw.com/static/index.html#/paint_index_v2). B) Heatmap of significantly different metabolites in T24 cells between the *siNC* and *siUSP52* groups. Metabolites associated with ferroptosis were labeled with red. C) Top 12 enriched pathways from integrated pathway analysis of significantly changed metabolites. Biosynthesis of unsaturated fatty acids, Glutathione metabolism, and Ferroptosis were showed in red. D) Heatmap of DEGs associated with ferroptosis in T24 cells between the *siNC* and *siUSP52* groups. E) qRT‐PCR analysis of DEGs associated with ferroptosis identified by RNA‐seq in *USP52*‐silenced T24 cells. F) BLCA cells were transfected with siRNAs or plasmids for 24 h. Then, 5000 transfected cells per well were seeded in the 96‐well plates for 24 h and treated with different concentrations of Era for 48 h. Cell viability of *USP52*‐knockdown (row 1) and USP52‐overexpressing cells (row 2) following treatment with different concentrations of Era were measured via MTT assay. G) T24 cells were transfected with indicated siRNAs (*siNC* or *siUSP52s*) for 24 h. Then, 5000 transfected cells per well were seeded in the 96‐well plates for 24 h and treated with 2 µm Era alone or in combination with 2 µm Ferrostatin‐1 (Fer‐1), 5 µm Z‐VAD, or 5 mm 3‐MA for 48 h. Cell viability of indicated groups was measured via MTT assay, and the absorbance was normalized to the Era‐ group. H) 5637 cells were transfected with indicated siRNAs (*siNC* or *siUSP52s*) for 24 h. Then, 5000 transfected cells per well were seeded in the 96‐well plates for 24 h and treated with 2 µm Era alone or in combination with 2 µm Fer‐1 for 48 h. Cell viability of indicated groups was measured via MTT assay, and the absorbance was normalized to the Era‐ group. I) Representative phase‐contrast images of *USP52*‐knockdown cells treated with 2 µm Era alone or in combination with 2 µm Fer‐1 for 48 h. Scale bar: 50 µm. J) T24 cells were transfected with indicated siRNAs (*siNC* or *siUSP52s*) for 24 h and treated with 2 µm Era alone or in combination with 2 µm Fer‐1 for 48 h. Then, collected and stained cells with pyridine iodide (PI) staining solution. Cell death rates (PI‐positive cells) were calculated via a flow cytometer. K) *USP52*‐depleted T24 cells were transfected with vector or USP52 plasmids for 24 h. Then, 5000 transfected cells per well were seeded in the 96‐well plates for 24 h and treated with 2 µm Era alone or in combination with 2 µm Fer‐1 for 48 h. Cell viability of indicated groups was measured via MTT assay, and the absorbance was normalized to the Era‐ group. L) T24 cells were transfected with indicated siRNAs (*siNC* or *siUSP52s*) for 24 h and treated with 2 µm Era for 24 h. Then, collected and stained cells with a BODIPY‐C11 probe. Lipid peroxidation was assessed by flow cytometry. M) T24 cells were transfected with vector or USP52 plasmids for 24 h and treated with 2 µm Era for 24 h. Then, collected and stained cells with a BODIPY‐C11 probe. Lipid peroxidation was assessed by flow cytometry. The data are presented as the means ± SDs, with *n *= 3 (B,D,E,J,L,M) or 4 (F–H,K) independent repeats. Statistical significance was determined by two‐tailed unpaired Student's *t‐*test (E) and one‐way ANOVA with Tukey's multiple comparisons (G,H,J–M).

In recent years, there has been growing recognition of the interconnectedness of various metabolic signaling pathways, including glutathione, cysteine, iron, and lipid metabolism pathways, ultimately leading to ferroptosis.^[^ ^28^ ^]^ To characterize the alterations in metabolites and genes induced by USP52, we performed gas chromatography‐mass spectrometry (GC‐MS) untargeted metabolomics and RNA sequencing (RNA‐seq) on T24 cells transfected with *siUSP52* (**Figure** **2**A). Heatmap analysis revealed significant changes in metabolic signatures following *USP52* knockdown (Figure 2B), including 8 ferroptosis‐related metabolites identified via the Kyoto Encyclopedia of Genes and Genomes (KEGG) compound database (https://www.kegg.jp/kegg/compound/). Particularly notable was the upregulation of arachidonic acid, which is known for its involvement in membrane phospholipids, especially phosphatidylethanolamine and phosphatidylcholine, which are crucial for lipid peroxidation and ferroptosis.^[^ ^29^ ^]^ Further enrichment analysis of these differentially abundant metabolites demonstrated that the biosynthesis of unsaturated fatty acids, glutathione metabolism, and ferroptosis pathways were significantly affected by *USP52* silencing (Figure 2C). In addition, numerous differentially expressed genes (DEGs) associated with ferroptosis were detected after *USP52* knockdown via RNA‐seq and subsequent qRT‐PCR confirmation (Figure 2D,E).

Functionally, the knockdown of *USP52* sensitized cancer cells to Erastin, while the overexpression of USP52 increased the resistance of cancer cells to Erastin (Figure 2F). A reduction in *USP52* significantly enhanced Erastin‐induced growth inhibition, which was fully reversed by the ferroptosis inhibitor Fer‐1 but not by inhibitors of other forms of RCD, including Z‐VAD‐FMK (an apoptosis inhibitor) and 3‐MA (an autophagy inhibitor) (Figure 2G,H). As judged by the flow cytometry analysis, depletion of *USP52* had a very minimal effect on cell apoptosis (apoptotic cell rates: 0.86% in *siNC* group, 2.84% in *siUSP52‐1* group, and 2.69% in *siUSP52‐2* group.) (Figure S2A, Supporting Information). Besides, alterations of proteins associated with cell apoptosis and autophagy were assessed by Western blot assay, which demonstrated that knockdown of *USP52* did not change the level of BAX and Bcl‐2 (apoptosis markers) and the level of p62 and LC3B (autophagy markers) (Figure S2B, Supporting Information). Recently, a mutually antagonistic relationship between ferroptosis and pyroptosis was revealed,^[^ ^30^ ^]^ which promoted us to explore whether USP52 was involved in the regulation of pyroptosis. As indicated in Figure S2B (Supporting Information), two pyroptosis regulators (GSDMD, cleaved caspase 1) exhibited no obvious change after *USP52* depletion. Therefore, the suppression of cell growth triggered by Erastin was evidently independent of apoptosis and autophagy upon downregulation of *USP52*. Consistently, increased cell death was observed in *USP52*‐depleted cells treated with Erastin, but these effects were reversed by treatment with Fer‐1 (Figure 2I,J). Moreover, the restoration of USP52 significantly reversed Erastin‐induced ferroptosis in T24 cells with stable depletion of *USP52* (Figure 2K; Figure S3A, Supporting Information). As lipid peroxidation is an important hallmark of ferroptosis, we next used the fluorescent probe BODIPY‐C11 to evaluate changes in lipid ROS in cells and found that *USP52* knockdown exacerbated lipid peroxidation, while USP52 overexpression prevented, Erastin‐induced lipid peroxidation in T24 cells (Figure 2L,M; Figure S3B,C, Supporting Information).

To further confirm that the loss of *USP52* renders cells susceptible to ferroptosis, we utilized tert‐butyl hydroperoxide (TBH) as a ROS inducer to stimulate ferroptosis. As shown in Figure S3D (Supporting Information), enforced overexpression of USP52 rendered bladder cancer cells resistant to TBH‐triggered ferroptosis, whereas knockdown of *USP52* sensitized cells to TBH‐triggered ferroptosis. Consistent with these findings, Fer‐1, rather than other RCD inhibitors, reversed the TBH‐induced growth delay in *USP52*‐deficient cells (Figure S3E,F, Supporting Information). Furthermore, the knockdown of *USP52* significantly aggravated TBH‐stimulated ferroptosis, which was fully restored by Fer‐1 treatment (Figure S3G,H, Supporting Information). Accordingly, the loss of *USP52* increased TBH‐induced lipid ROS in UM‐UC‐3 cells (Figure S3I,J, Supporting Information). In contrast, USP52 overexpression notably promoted the detoxification of TBH‐induced lipid peroxidation (Figure S3K,L, Supporting Information). Taken together, our findings indicate that the loss of *USP52* determines tumor cell sensitivity to ferroptosis in BLCA.

### 2.3 USP52 Promotes BLCA Proliferation In Vitro

**Figure 3:**
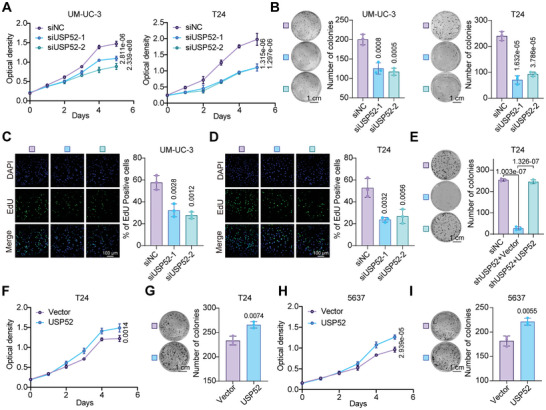
USP52 promotes cell proliferation in bladder cancer cells. A,F,H) BLCA cells were transfected with indicated siRNAs or plasmids for 48 h. Then, 2000 transfected cells per well were seeded in the 96‐well plates, followed by an assessment of cell viability with MTT assay for five consecutive days. Cell proliferation curves of the indicated cells with *USP52* knockdown (A) or overexpression (F,H) were showed. B,G,I) BLCA cells were transfected with indicated siRNAs or plasmids for 48 h. Then, 1000 transfected cells per well were seeded and cultured in the 6‐well plates for 9–12 days, followed by fixation, staining, and photograph. Representative images (left panel) and statistical graph (right panel) of colony formation assays from the indicated groups with *USP52* knockdown (B) or USP52 overexpression G,I) were showed. Scale bar: 1 cm. C,D) BLCA cells were transfected with indicated siRNAs for 48 h in the 6‐well plates covered with a cover glass. Then, transfected cells were fixed and stained with an EdU staining kit. Images of EdU‐positive cells were captured with a confocal microscope. Representative images (left panel) and statistical graphs (right panel) of EdU staining assays from the indicated groups of UM‐UC‐3 (C) and T24 (D) cells with *USP52* knockdown were showed. Blue indicated nuclear staining and green indicated EdU‐positive cells. EdU‐positive rates were calculated by dividing the number of EdU‐positive cells by the number of cells stained with nuclear. Scale bar: 100 µm. E) *USP52*‐depleted T24 cells were transfected with vector or USP52 plasmids for 48 h. Then, 1000 transfected cells per well were seeded and cultured in the 6‐well plates for 9–12 days, followed by fixation, staining, and photograph. Representative images (left panel) and statistical graph (right panel) of colony formation assays showing *USP52*‐depleted cells with or without USP52 re‐expression. Scale bar: 1 cm. The data are presented as the means ± SDs, with *n* = 3 (B‐E, G, I) or 5 (A,F,H) independent repeats. Statistical significance was determined by two‐tailed unpaired Student's *t*‐test (F–I) or one‐way ANOVA with Tukey's multiple comparisons (A–E).

MTT assays revealed that knockdown of *USP52* in T24 and UM‐UC‐3 cells significantly inhibited cell growth (**Figure** **3**A). Additionally, silencing *USP52* significantly attenuated anchorage‐dependent growth in T24 and UM‐UC‐3 cells, as evidenced by the colony formation assay (Figure 3B). To assess cell proliferation, we utilized 5‐ethynyl‐2′‐deoxyuridine (EdU), a thymidine analog that is incorporated into cellular DNA during DNA synthesis.^[^ ^31^ ^]^ As expected, *USP52* knockdown markedly decreased the percentage of EdU‐positive T24 and UM‐UC‐3 cells (Figure 3C,D). Again, Fer‐1 partially rescued *USP52* deletion–induced growth suppression, but apoptosis inhibitor Z‐VAD or pyroptosis inhibitor Disulfiram did not, indicating that the depletion of *USP52* inhibited cell growth in a ferroptosis‐dependent manner (Figure S4, Supporting Information). In addition, the re‐expression of USP52 in stably USP52‐deficient T24 cells completely restored the inhibitory effect of *USP52* depletion on cellular proliferation (Figure 3E). Furthermore, overexpression of USP52 promoted cell proliferation in both T24 and 5637 cells, as evidenced by the MTT and colony formation assays (Figure 3F–I). Overall, *USP52* ablation inhibits BLCA proliferation in cultured cells.

### 2.4 Loss of *USP52* Impairs Cell Proliferation by Decreasing xCT and Promoting Ferroptosis

**Figure 4:**
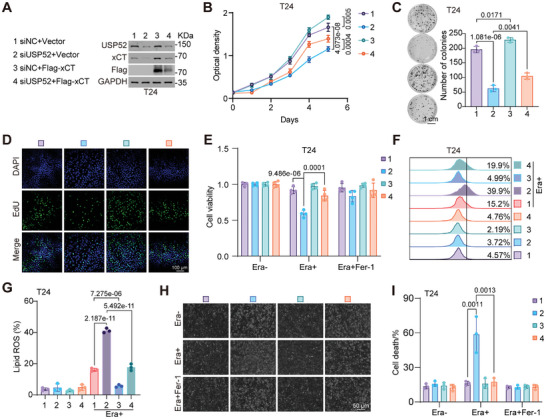
Depletion of *USP52* inhibits bladder cancer progression by decreasing xCT and promoting ferroptosis. A) T24 cells were transfected with indicated siRNAs (*siNC* and *siUSP52*) for 24 h, followed by transfection with a vector of Flag‐xCT plasmids for 48 h. Four groups in this Figure were indicated as 1, 2, 3, and 4. Cells were collected and lysed for total protein extraction. Western blot assays showing the expression of USP52 and xCT in *USP52*‐silenced T24 cells with or without xCT re‐expression. B) About 2000 transfected cells per well were seeded in the 96‐well plates, followed by an assessment of cell viability with MTT assay for five consecutive days. Cell proliferation curves of *USP52*‐silenced T24 cells with or without xCT re‐expression were showed. C) About 1000 transfected cells per well were seeded and cultured in the 6‐well plates for 9–12 days, followed by fixation, staining, and photograph. Representative images (left panel) and statistical graph (right panel) of colony formation assays of *USP52*‐silenced T24 cells with or without xCT re‐expression were showed. D) Transfected T24 cells were fixed and stained with an EdU staining kit. Images of EdU‐positive cells captured by a confocal microscope in *USP52*‐silenced cells with or without xCT re‐expression were showed. Blue indicated nuclear staining and green indicated EdU‐positive cells. E) About 5000 transfected T24 cells per well were seeded in the 96‐well plates for 24 h and treated with 2 µm Era alone or in combination with 2 µm Fer‐1 for 48 h. Cell viability of indicated groups were measured via MTT assay, and the absorbance was normalized to the Era‐ group. F,G) Transfected T24 cells were treated with 2 µm Era for 24 h. Then, collected and stained cells with a BODIPY‐C11 probe. Lipid peroxidation was assessed by flow cytometry. Representative data (F) and statistical graph (G) of lipid ROS in T24 cells with indicated groups were showed. H) Representative phase‐contrast images of transfected T24 cells treated with 2 µm Era alone or in combination with 2 µm Fer‐1 for 48 h. I) Transfected T24 cells were treated with 2 µm Era alone or in combination with 2 µm Fer‐1 for 48 h. Then, collected and stained cells with pyridine iodide (PI) staining solution. Cell death rates (PI‐positive cells) were calculated via a flow cytometer. Scale bars: 1 cm (C), 100 µm (D) and 50 µm (H). The data are presented as the means ± SDs, with *n* = 3 (C,G,I) or 4 (E) or 5 (B) independent repeats. Statistical significance was determined by one‐way ANOVA with Tukey's multiple comparisons (B,C,E,G,I).

We then induced the transfection‐mediated overexpression of xCT in both T24 and UM‐UC‐3 cells following *USP52* knockdown (**Figure** **4**A; Figure S5A, Supporting Information). MTT assays showed that overexpression of xCT alone significantly promoted cell proliferation, and restoration of xCT in transiently silenced *USP52* cells reversed the inhibitory effect on cell growth induced by knockdown of *USP52* (Figure 4B; Figure S5B, Supporting Information). Similar phenotypes were observed in the colony formation and EdU staining assays (Figure 4C,D; Figure S5C,D, Supporting Information). These results revealed that overexpression of xCT patially rescued the loss of *USP52*‐induced suppression of cell proliferation. To determine whether the reduction in *USP52*‐promoted ferroptosis depended on xCT, we transfected exogenous xCT plasmids into *USP52*‐knockdown cells with or without ferroptosis inducers and inhibitors. Notably, knockdown of *USP52* markedly promoted Erastin‐ and ROS‐triggered ferroptosis, which was largely rescued by xCT restoration, as evidenced by cell viability, lipid ROS levels, and cell death assays in both T24 and UM‐UC‐3 cells (Figure 4E–I; Figure S5E–I, Supporting Information). Overall, these findings collectively suggest that the loss of *USP52* suppresses BLCA progression by impairing xCT expression and enhancing sensitivity to ferroptosis in vitro.

### 2.5 USP52 Physically Interacts with xCT and Maintains Its Stability

**Figure 5:**
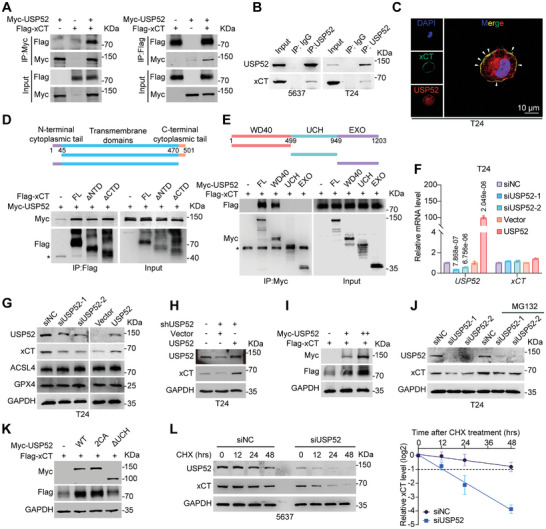
USP52 interacts with xCT and maintains its protein stability. A) HEK293T cells were transfected with indicated plasmids for 48 h. Western blot analysis of Myc‐USP52 and Flag‐xCT after Myc‐IP or Flag‐IP in HEK293T cells were showed. The input was 10% of the extract used for the IP. B) Western blot analysis of USP52 and xCT after USP52‐IP or IgG‐IP in 5637 and T24 cells. The input was 10% of the extract used for the IP. C) Confocal microscopy images of immunofluorescence staining of USP52 (red) and xCT (green) in T24 cells. The white arrow indicated the colocalization of USP52 and xCT in the cell membrane. Scale bar: 10 µm. D) Schematic diagram of various xCT truncations in the Co‐IP assays (top panel). HEK293T cells were transfected with indicated plasmids for 48 h. Western blot analysis of Myc‐USP52 and Flag‐xCT after Flag‐IP in HEK293T cells (bottom panel). ^*^: indicated IgG heavy chain. E) Schematic diagram of various USP52 truncations in the Co‐IP assays (top panel). HEK293T cells were transfected with indicated plasmids for 48 h. Western blot analysis of Myc‐USP52 and Flag‐xCT after Myc‐IP in HEK293T cells (bottom panel). ^*^: indicated IgG heavy chain. F) T24 cells were transfected with indicated siRNAs or plasmids for 48 h, followed by a collection for qRT‐PCR assay. The relative mRNA levels of *USP52* and *xCT* in *USP52*‐silenced or USP52‐overexpressing T24 cells were showed. G) T24 cells were transfected with indicated siRNAs or plasmids for 48 h, followed by a collection for Western blot assay. The protein levels of ferroptosis‐related proteins in T24 cells with *USP52* knockdown or USP52 overexpression were showed. H) *USP52*‐depleted T24 cells were transfected with vector or USP52 plasmids for 48 h, followed by a collection for Western blot assay. The protein levels of USP52 and xCT in *USP52*‐depleted T24 cells with or without USP52 re‐expression were showed. I) HEK293T cells were transfected with Flag‐xCT (1 µg) alone or in combination with increasing amounts of Myc‐USP52 (1, 3 µg) for 48 h, followed by a collection for Western blot assay. The protein levels of Myc‐USP52 and Flag‐xCT were showed. J) T24 cells were transfected with indicated siRNAs (*siNC* and *siUSP52*s) for 48 h, followed by treatment with 10 µm 6 h before collection. Western blot analysis of USP52 and xCT in *USP52*‐silenced T24 cells with or without 10 µm MG132 treatment were showed. K) HEK293T cells were transfected with Flag‐xCT alone or in combination with and Myc‐USP52‐WT, Myc‐USP52‐2CA, and Myc‐USP52‐ΔUCH plasmids for 48 h, followed by a collection for Western blot assay. The protein levels of Myc‐USP52 and Flag‐xCT in the indicated groups were showed. L) 5637 cells were transfected with indicated siRNAs (*siNC* and *siUSP52*) for 48 h and treated with 50 µg mL^−1^ CHX as indicated hours, followed by collection for Western blot assay. Representative Western blot images showing the effect of *USP52* knockdown on xCT degradation in 5637 cells (left panel) and a statistical diagram of the results of the protein half‐life assays (right panel). The data are presented as the means ± SDs, with *n* = 3 (F,L) independent repeats. Statistical significance was determined by one‐way ANOVA with Tukey's multiple comparisons (F).

To better understand the underlying mechanism of the USP52‐xCT axis in the regulation of BLCA progression, we further performed a series of coimmunoprecipitation (co‐IP) analyses. Plasmids encoding Myc‐tagged USP52 and Flag‐tagged xCT were transfected into HEK293T cells, and co‐IP assays revealed an exogenous interaction between USP52 and xCT (**Figure** **5**A). Similarly, endogenous USP52 efficiently interacted with xCT in 5637 and T24 cells (Figure 5B). Subsequently, confocal microscopy analysis further demonstrated the colocalization of USP52 with xCT in the membrane of T24 cells (Figure 5C). To identify the specific domains responsible for the association between USP52 and xCT, we constructed a panel of truncated mutant plasmids and transfected them into HEK293T cells. Next, co‐IP analysis revealed that the N‐terminal domain, transmembrane domain, and C‐terminal domain of xCT were indispensable for its association with USP52 (Figure 5D), while the N‐terminal WD40 domain of USP52 was sufficient and necessary for its interaction with xCT (Figure 5E).

As USP52 is a DUB, we next examined whether USP52 affects the protein abundance of xCT. As shown in Figure 5F, neither knockdown nor overexpression of USP52 had a minimal effect on the transcriptional level of xCT, suggesting regulation at the translational level. As hypothesized, knockdown of *USP52* significantly downregulated the protein level of xCT, while no obvious changes were observed in the levels of ACSL4 and GPX4, two well‐known regulators of ferroptosis^[^ ^32^ ^]^ (Figure 5G; Figure S6A, Supporting Information). Conversely, overexpression of USP52 increased xCT protein levels but had no effect on ACSL4 or GPX4 in either T24 or UM‐UC‐3 cells (Figure 5G; Figure S6A, Supporting Information). Interestingly, we concerned a mRNA‐protein expression level discrepancy that knockdown of *USP52* led to a downregulation of ACSL4 at mRNA level (Figure 2D,E) but had no effect on ACSL4 protein (Figure 5G; Figure S6A, Supporting Information). This discrepancy might due to that gene expression is intensively regulated by multiple‐level processes, including transcriptional regulation and post‐transcriptional regulation, to maintain a cellular state or adapt to the environment. Intuitively, the post‐transcriptional and translational regulation processes should give rise to a major difference between mRNA and protein levels.

To further investigate the regulatory relationship between USP52 and xCT at the protein level, we mixed *siNC* and *siUSP52* BLCA cells and then performed immunofluorescence staining assays, which revealed that knockdown of *USP52* efficiently decreased the protein level of xCT in T24 and UM‐UC‐3 cells (Figure S6B, Supporting Information). Furthermore, the restoration of USP52 in T24 cells with stable depletion of *USP52* reversed the decrease in xCT protein levels caused by the loss of *USP52* (Figure 5H). Notably, overexpression of USP52 in HEK293T cells increased the protein level of exogenous Flag‐tagged xCT in a dose‐dependent manner (Figure 5I). Moreover, the decrease in the xCT protein level associated with *USP52* knockdown was effectively counteracted by treatment with the proteasome inhibitor MG132, indicating that *USP52* depletion‐mediated degradation of xCT occurs via the ubiquitin‐proteasome pathway (Figure 5J; Figure S6C, Supporting Information).

To further explore whether the enzymatic catalytic activity of USP52 regulates xCT protein abundance, we overexpressed wild‐type USP52, USP52‐2CA (C528A/C530A mutants, an important but not essential for USP52 catalysis mutants reported by Yang et al.^[^ ^18b^ ^]^), or USP52‐ΔUCH (lacking its catalytic domain UCH mutants) along with xCT in HEK293T cells. Interestingly, both wild‐type USP52 and USP52‐2CA, but not USP52‐ΔUCH, significantly increased the protein level of exogenous xCT (Figure 5K). Similarly, as shown in Figure S6D (Supporting Information), wild‐type USP52 and USP52‐2CA promoted the protein abundance of endogenous xCT in a dose‐dependent manner, while USP52‐ΔUCH had minimal effect on xCT protein expression in T24 cells. In addition, cycloheximide (CHX) chase assays in 5637 cells demonstrated that the loss of function of USP52 effectively shortened the half‐life of xCT (Figure 5L), while the half‐life of xCT was longer in USP52‐overexpressing cells than in control cells (Figure S6E, Supporting Information). Altogether, these findings indicate that USP52 interacts with xCT and promotes its stability in an enzyme‐dependent manner.

### 2.6 USP52 Deubiquitinates xCT at K4 and K12

**Figure 6:**
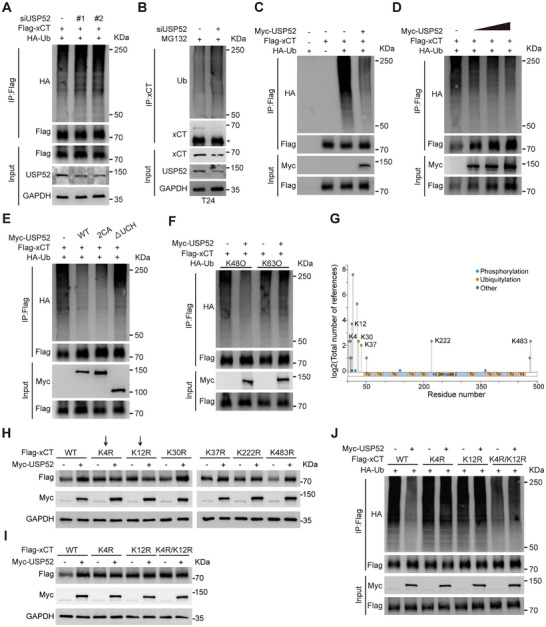
USP52 deubiquitinates xCT at K4 and K12. A) HEK293T cells were transfected with Flag‐xCT and HA‐Ub together with control siRNA or *USP52* siRNA as indicated for 72 h and then treated with 10 µm MG132 for 6 h. Cellular extracts were prepared for IP assays after Flag‐IP followed by immunoblotting with anti‐HA. B) T24 cells were transfected with control siRNA or *USP52* siRNA for 48 h and then incubated with 10 µm MG132 for 6 h. IP assays were performed to measure the level of the ubiquitin conjugates of xCT after xCT‐IP, followed by immunoblotting with an anti‐ubiquitin antibody. ^*^: indicated IgG heavy chain. C) HEK293T cells were transfected with the described plasmids as indicated for 48 h and then treated with 10 µm MG132 for 6 h. Cellular extracts were prepared for IP assays after Flag‐IP followed by immunoblotting with anti‐HA. D) HEK293T cells were transfected with Flag‐xCT and HA‐Ub together with empty control or increasing amounts of Myc‐USP52 (1, 3, and 6 µg) as indicated for 48 h and then treated with 10 µm MG132 for 6 h. Cellular extracts were prepared for IP assays after Flag‐IP followed by immunoblotting with anti‐HA. E) HEK293T cells were transfected with Flag‐xCT and HA‐Ub together with empty control or Myc‐USP52 mutants as indicated for 48 h and then treated with 10 µm MG132 for 6 h. Cellular extracts were prepared for IP assays after Flag‐IP followed by immunoblotting with anti‐HA. F) HEK293T cells were transfected with Flag‐xCT and HA‐Ub (K48O or K63O) together with empty control or Myc‐USP52 as indicated for 48 h and then treated with 10 µm MG132 for 6 h. The IP assays were performed after Flag‐IP followed by immunoblotting with anti‐HA. G) Ubiquitination sites of xCT predicted by the PhosphoSitePlus database. The *X*‐axis represented the residue number of human xCT protein and the *Y*‐axis represented a total number of references. H) HEK293T cells were transfected with 2 µg wild‐type Flag‐xCT or six K‐to‐R mutants together with empty control or Myc‐USP52 for 48 h in the first round of screening, followed by collection for Western blot assay. The expression of Flag‐xCT detected with an anti‐Flag antibody was showed. I) HEK293T cells were transfected with 2 µg wild‐type Flag‐xCT, K4R, K12R, or K4R/K12R mutants together with empty control or Myc‐USP52 for 48 h in the second round of screening, followed by a collection for Western blot assay. The expression of Flag‐xCT detected with an anti‐Flag antibody were showed. J) HEK293T cells were transfected with wild‐type Flag‐xCT or different K‐to‐R mutants and HA‐Ub together with empty control or Myc‐USP52 as indicated for 48 h and then treated with 10 µm MG132 for 6 h. The IP assays were performed after Flag‐IP followed by immunoblotting with anti‐HA.

Subsequently, we investigated whether USP52 stabilized xCT through deubiquitination. An in vivo ubiquitination assay showed that the knockdown of *USP52* effectively increased the level of ubiquitinated xCT (**Figure** **6**A). In addition, the conjugation of endogenous ubiquitin to xCT significantly increased after *USP52* depletion (Figure 6B). In contrast, overexpression of USP52 led to a dramatic reduction in xCT ubiquitylation in a dose‐dependent manner (Figure 6C,D; Figure S7A, Supporting Information). Similarly, USP52‐2CA retained the ability to remove ubiquitin linkages from xCT, whereas USP52‐ΔUCH mutants exhibited almost no effect on xCT ubiquitination (Figure 6E). We further investigated the specific type of ubiquitin chains on xCT cleaved by USP52 and found that USP52 removed K48‐linked ubiquitin chains from xCT but not K6‐, K11‐, K27‐, K29‐, K33‐ and K63‐linked chains (Figure 6F; Figure S7B, Supporting Information).

These findings prompted us to identify the ubiquitin conjugation sites on xCT. As shown in Figure 6G, using the online bioinformatics database PhosphoSitePlus (http://www.phosphosite.org/), we identified a total of six putative ubiquitination sites (K4, K12, K30, K37, K222. K483). Then, we individually mutated these lysine (K) residues to arginine (R) residues and coexpressed them with Myc‐USP52. Ectopic expression of USP52 significantly increased the protein level of all xCT point mutants except for K4R and K12R (Figure 6H; Figure S7C,E,G, Supporting Information). Consequently, we constructed a K4R/K12R double point mutant of xCT, and our results indicated that USP52 overexpression failed to upregulate the xCT protein with K4R/K12R mutations (Figure 6I; Figure S7D,F,G, Supporting Information). Furthermore, USP52 effectively attenuated the ubiquitination level of wild‐type xCT but not that of xCT mutants, including K4R, K12R, and K4R/K12R (Figure 6J). In conclusion, USP52 acts as a bona fide DUB of xCT, targeting K4 and K12 of xCT to cleave K48‐linked ubiquitination.

### 2.7 Loss of *USP52* Represses BLCA Progression by Promoting Ferroptosis In Vivo

**Figure 7:**
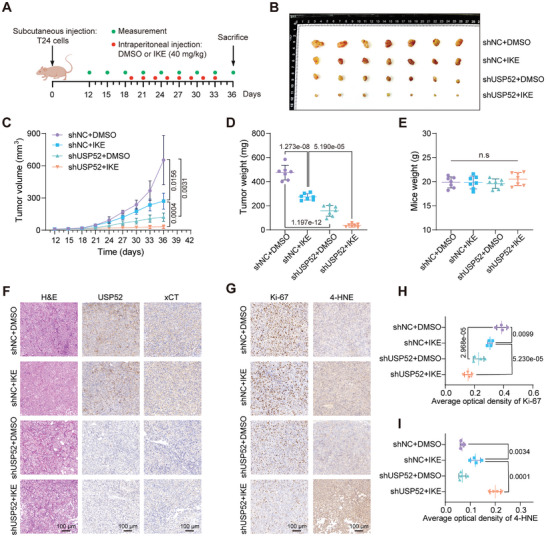
Loss of *USP52* suppresses BLCA progression by promoting ferroptosis in vivo. A) Schematic diagram for evaluating the therapeutic effects of USP52 in a bladder cancer xenograft model. The progression of subcutaneous tumors was monitored three days for day 12 until the endpoint. This Figure was drawn by an online tool Figdraw (https://www.figdraw.com/static/index.html#/paint_index_v2). B) Images of xenograft tumors that were inoculated into nude mice with the indicated T24 cell lines for 36 days. C) Volumes of xenograft tumors in the indicated groups on different days. D) Weights of xenograft tumors in the indicated groups at the endpoint. E) Body weight of mice in the indicated groups at the endpoint. F) Representative images of hematoxylin and eosin (H&E) (left panel) and IHC staining (right panel: USP52 and xCT) of tumor xenografts from the indicated groups. G) Representative images of IHC staining for Ki‐67 and 4‐HNE in tumor xenografts from the indicated groups. H,I) The average optical density of proteins was assessed with ImageJ software. Statistical graphs of IHC staining for Ki‐67 (H) and 4‐HNE (I) in tumor xenografts from the indicated groups. Scale bar: 100 µm. The data are presented as the means ± SDs, with *n* = 4 (H,I) or 7 (C–E) independent repeats. Statistical significance was determined by one‐way ANOVA with Tukey's multiple comparisons (D,E,H,I) or two‐way ANOVA with Tukey's multiple comparisons (C).

We next explored the therapeutic potential of *USP52* depletion to enhance sensitivity to ferroptosis in vivo. As shown in the flow graphs, shNC or shUSP52 T24 cells were subcutaneously injected into the mice to generate xenograft tumors. The mice were then treated with either 40 mg kg^−1^ imidazole ketone Erastin (IKE, a potent, metabolically stable, selective system x_c_^−^ inhibitor, or ferroptosis inducer^[^ ^33^ ^]^) or DMSO every other day for two weeks starting on day 19 (**Figure** **7**A). Mice treated with IKE alone exhibited slowed tumor growth, as evidenced by measurements of tumor size and weight (Figure 7B–D). In addition, the loss of *USP52* resulted in an evident decrease in tumor growth (Figure 7B–D). Notably, the combination of *USP52* depletion and IKE administration dramatically inhibited tumor growth without any detectable side effects on the mice, as evidenced by no significant changes in mouse weight observed across the four groups (Figure 7B–E). Immunohistochemical (IHC) staining analysis further confirmed that *USP52* knockdown contributed to a marked reduction in the xCT protein level in vivo (Figure 7F). Consistently, treatment with IKE or *USP52* depletion largely decreased the average optical density of Ki‐67, an effective marker of proliferation, and this suppressive effect was further significantly potentiated by shUSP52 combined with IKE treatment (Figure 7G,H). The level of 4‐hydroxynonenal (4‐HNE), a stable product of lipid peroxidation and a ferroptosis indicator,^[^ ^34^ ^]^ was obviously increased after IKE treatment (Figure 7G,I). In addition, the loss of *USP52* significantly increased the average optical density of 4‐HNE after IKE administration (Figure 7G,I). These observations provided strong evidence that *USP52* depletion sensitizes tumor cells to ferroptosis. Thus, combined *USP52* depletion and ferroptosis induction synergistically suppressed BLCA progression.

### 2.8 USP52 is Associated with BLCA Progression and Prognosis

**Figure 8:**
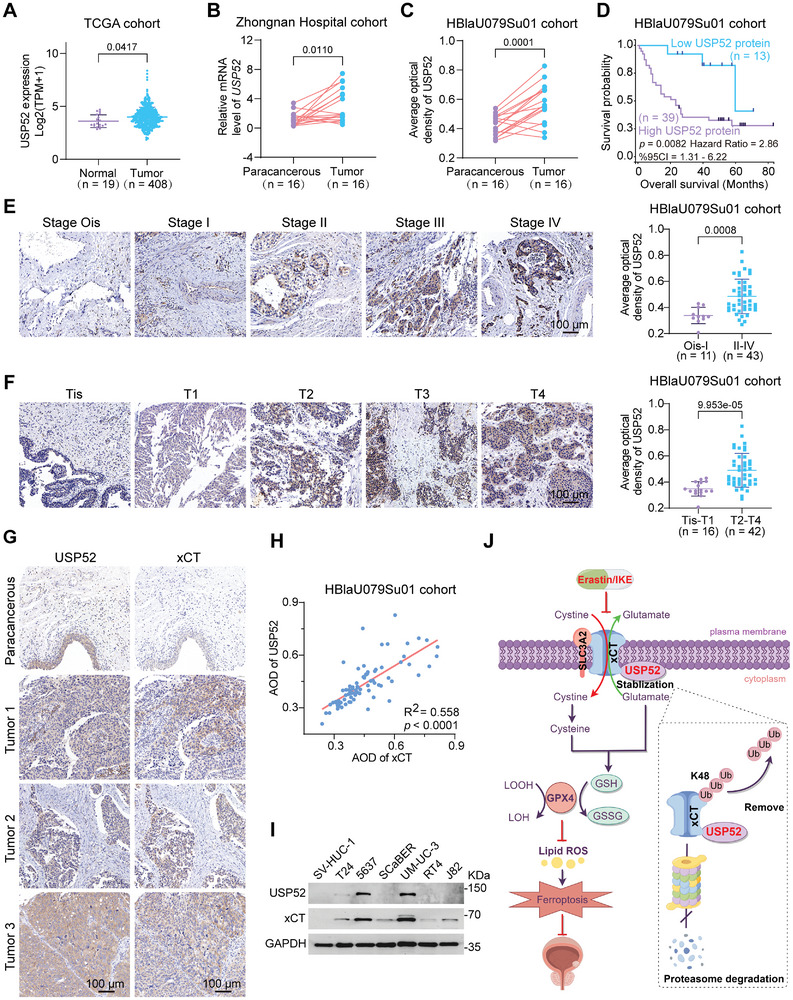
USP52 is positively correlated with xCT and is associated with BLCA progression and prognosis. A) The mRNA level of *USP52* in BLCA and normal tissues in the TCGA‐BLCA cohort (RNA‐seq data). TPM: transcripts per million kilobases. B) 16 pairs of matched BLCA and paracancerous tissues were collected for qRT‐PCR assay form Zhongnan Hospital. The relative mRNA level of *USP52* in BLCA and paracancerous tissues in the Zhongnan Hospital cohort were showed. C) The average optical density of the USP52 protein in 16 pairs of matched BLCA and paracancerous tissues from the HBlaU079Su01 cohort were analyzed via IHC staining. D) The prognostic curve (overall survival) of patients with different USP52 protein levels in the HBlaU079Su01 cohort was analyzed. The patients were divided into high and low USP52 protein level groups according to the quartile USP52 expression. E) Representative images (left panel) and statistical graphs (right panel) of IHC staining analysis of USP52 protein levels in different stages (the seventh edition of the AJCC: stage Ois, stage I, stage II, stage III, and stage IV) from the HBlaU079Su01 cohort. F) Representative images (left panel) and statistical graphs (right panel) of the results of the IHC staining analysis of USP52 protein levels at various T stages (Tis, T1, T2, T3, and T4) in samples from the HBlaU079Su01 cohort. G) Representative IHC images of USP52 and xCT in four identical tissues (1 paracancerous and 3 BLCA tissues) from the HBlaU079Su01 cohort. H) The correlation between USP52 and xCT protein expression in the HBlaU079Su01 cohort was analyzed by IHC staining. The average optical density of proteins was assessed with ImageJ software. The correlation coefficient and *p*‐value were showed. I) 1 normal uroepithelial cell line (SV‐HUC‐1) and 6 BLCA cell lines (T24, 5637, SCaBER, UM‐UC‐3, RT4, and J82) were collected for Western blot assay. The protein expression of USP52 and xCT in multiple cell lines was showed. J) Mechanism diagram of this study. This Figure was drawn by an online tool Figdraw (https://www.figdraw.com/static/index.html#/paint_index_v2). Scale bar: 100 µm. The data are presented as the means ± SDs. Statistical significance was determined by two‐tailed unpaired Student's *t*‐test (A,E,F), two‐tailed paired Student's *t*‐test (B,C), Pearson's correlation (H), or the log‐rank test of Kaplan–Meier analysis (D).

All the aforementioned findings regarding the regulation and underlying mechanism of the USP52‐xCT axis prompted us to further investigate whether USP52 is linked to BLCA progression and prognosis in clinical samples. USP52 is commonly upregulated in BLCA according to The Cancer Genome Atlas (TCGA) database (**Figure** **8**A; Figure S8A, Supporting Information). Furthermore, we collected 16 pairs of human BLCA and paracancerous specimens from our hospital and confirmed that the mRNA level of *USP52* was significantly increased in the tumor samples (Figure 8B, Table S1, Supporting Information). Notably, quantitative analysis of IHC in a BLCA tissue microarray (TMA) indicated an evident increase in the average optical density of USP52 in tumor tissues compared to that in paracancerous tissues (Figure 8C).

In the TMA cohort (HBlaU079Su01), patients with high USP52 protein expression had worse overall survival outcomes than those with low USP52 protein expression (Figure 8D). Additionally, the protein level of USP52 showed a positive correlation with the ATCC stage and T stage of BLCA in our TMA cohort (Figure 8E,F, Table S2, Supporting Information). Importantly, xCT was also highly expressed in BLCA, and the protein levels of xCT and USP52 exhibited strong positive associations with each other (Figure 8G,H). Consistently, similar results were observed in the normal uroepithelial cell line (SV‐HUC‐1) and in the BLCA cell lines (T24, 5637, SCaBER, UM‐UC‐3, RT4, and J82) (Figure 8I; Figure S8B, Supporting Information) as judged by Western blot assay. Furthermore, the mRNA levels of USP52 and xCT in these cell lines were investigated. The mRNA level of USP52 was increased in ScaBER, UM‐UC‐3, and J82 when compared to SV‐HUC‐1. Besides, the mRNA level of xCT in these BLCA cell lines was observably increased (Figure S8C, Supporting Information). Overall, we demonstrated a strong correlation between USP52 and the progression and prognosis of BLCA patients. In addition, our findings indicated a positive association between USP52 and xCT in both clinical samples and bladder cancer cell lines.

Thus, USP52 was identified as a bone fide DUB of xCT that physically interacts with xCT and biochemically cleaves the K48‐conjugated ubiquitin chains of xCT at K4 and K12, thereby maintaining its stability. Loss of *USP52* suppressed cell proliferation by inhibiting xCT expression and sensitizing tumor cells to ferroptosis. Moreover, the combination of *USP52* depletion and IKE synergistically inhibited the progression of BLCA by inducing ferroptosis, suggesting a potential novel therapeutic strategy for treating BLCA (Figure 8J).

## 3 Discussion

Since ferroptosis was first defined in 2012,^[^ ^3a^ ^]^ extensive research has been conducted on its regulatory mechanisms and potential roles in tumorigenesis. Ferroptosis has been reported to inhibit tumorigenesis in different models, for example, lung cancer,^[^ ^35^ ^]^ colorectal cancer,^[^ ^36^ ^]^ and hepatocellular carcinoma,^[^ ^37^ ^]^ which has potential as an innovative strategy for therapeutic targeting.^[^ ^38^ ^]^ In the context of BLCA, few studies have elucidated the specific role of ferroptosis, particularly focusing on the xCT protein and the underlying mechanisms by which it affects disease progression. For example, low pathological stage BLCA cells were highly sensitive to RSL3‐induced ferroptosis in vitro.^[^ ^39^ ^]^ In addition, Liang et al.^[^ ^40^ ^]^ reported that xCT was overexpressed in BLCA and that its high level was associated with worse survival in BLCA patients. Consistently, our study revealed an increase in xCT protein in BLCA, as judged by the IHC staining of a TMA. By reanalyzing our scRNA sequencing data, we first revealed that BLCA patients with a high ferroptosis state exhibited a lower T stage at the single cell level, which suggested that ferroptosis might exert a suppressive effect on BLCA progression in clinical. Despite these encouraging observations, whether ferroptosis, especially this induced by block xCT‐mediated functional activity, plays an inhibitory role on BLCA progression remains elusive.

Erastin, first discovered in 2003,^[^ ^41^ ^]^ is a commonly used ferroptosis inducer. Due to its instability and low solubility in vivo, Erastin is unsuitable for use in animal studies.^[^ ^42^ ^]^ IKE serves as a viable alternative to Erastin used in animal studies due to its metabolic stability and increased solubility.^[^ ^43^ ^]^ In a diffuse large B‐cell lymphoma xenograft model, IKE suppressed tumor growth by promoting ferroptosis.^[^ ^43^ ^]^ In this study, IKE treatment also exhibited antitumor activity in our BLCA xenograft model without any side effects, which further verified a tumor suppressor role of ferroptosis in BLCA in vivo. Recent studies have discovered various ferroptosis inducers, some of which have been used for tumor therapy in clinical, for instance, sorafenib, which functions to restrain xCT activity.^[^ ^42^ ^]^ Even so, research on strategies for therapeutic ferroptosis induction must be carefully evaluated to identify optimal compounds, doses, and schedules, as well as tumor indications in the future.

Identification and in‐depth understanding of specific vulnerabilities in BLCA cells that make them sensitive to ferroptosis provides an opportunity for more precise medical treatment of BLCA. Thereout, by screening out an unbiased DUBs RNAi library, USP52/PAN2 was identified as a novel DUB involved in ferroptosis sensitivity and xCT protein regulation. USP52, previously known as a pseudodeubiquitinase, binds to PAN3 to regulate mRNA stability through deadenylation.^[^ ^44^ ^]^ It was reported that USP52 is an important component of P‐bodies that is required to prevent HIF1A mRNA degradation under hypoxic conditions.^[^ ^45^ ^]^ USP52 was identified as a true DUB that stabilizes the histone chaperone ASF1A by removing K48‐like polyubiquitin chains through its UCH domain, which in turn facilitates chromatin assembly, favors cell cycle progression and promotes breast carcinogenesis.^[^ ^18b^ ^]^ In addition, USP52 is involved in the regulation of DNA end resection and chemosensitivity through direct interaction with and deubiquitination of CtIP.^[^ ^19^ ^]^

Here, we uncovered an evident increase in the expression of USP52 at transcriptional and translational levels in BLCA tissues compared to that in paracancerous tissues. Besides, patients with high USP52 protein expression had worse overall survival outcomes. Functionally, the loss of *USP52* inhibited BLCA progression by repressing xCT expression and sensitizing tumor cells to ferroptosis. Besides, the antitumor activity of USP52 depletion alone was significantly enhanced by ferroptosis induction in vivo, which suggested a sound therapeutic strategy of BLCA patients with high USP52 levels.

Mechanistically, USP52 physically associated with xCT through its WD40 domain, leading to xCT K48‐conjugated deubiquitination at K4 and K12, which in turn promoted xCT protein stability. Recently, it was reported that USP20 directly interacts with xCT and cleaves K48‐linked polyubiquitinated xCT at K30 and K37.^[^ ^46^ ^]^ TRIM3, an E3 ubiquitin ligase, promoted xCT proteasome‐dependent degradation by catalyzing K11‐linked ubiquitination of xCT at K37.^[^ ^47^ ^]^ Notably, no reports have identified any E3 ligase‐mediated ubiquitination of the xCT protein at K4 or K12, leaving an open question regarding the identification of another antagonistic regulator responsible for ferroptosis sensitivity and ubiquitination modification of xCT at K4 or K12 in BLCA in future studies. Because several deubiquitinases, such as USP18, DUBA, and OTUD5, have recently been reported as deubiquitinase for xCT,^[^ ^48^ ^]^ it will be encouraging to study whether xCT is also dynamically regulated by these DUBs in BLCA. To ensure the proper function of xCT‐mediated cystine transport and subsequent redox balance, the expression and activity of xCT are strictly regulated by several types of posttranslational modifications (PTMs).^[^ ^37^, ^49^ ^]^ Palmitoylation, as another one of the most common PTMs, might promote downstream protein degradation by affecting protein ubiquitination,^[^ ^50^ ^]^ and xCT can be palmitoylated by ZDHHC8 at C327.^[^ ^49a^ ^]^ Based on this, it will be interesting to investigate whether or under what conditions USP52‐modified xCT deubiquitination is influenced by ZDHHC8‐mediated palmitoylation at C327.

Overall, with respect to cultured cell lines, animal tumor‐bearing models, and clinical human samples, we revealed that USP52 controlled the sensitivity of tumor cells to ferroptosis by stabilizing the xCT protein in a deubiquitinase‐dependent manner, which consequently facilitated BLCA progression. Genetic blockade of USP52 and/or IKE might be a potential strategy for developing BLCA‐targeting therapies.

## 4 Experimental Section

### Single‐Cell RNA Analysis

scRNA data was generated in a previous study.^[^ ^22^ ^]^ The epithelial cells were extracted from the total integrated scRNA dataset. Ferroptosis gene scoring was performed with Seurat::AddModuleScore (v4.0.1) in R (v4.1.3). Only treatment‐naive BLCA tissues were used to compute the prevalence of ferroptosis‐high and low epithelial cells.

### Human BLCA Tissues

Human BLCA and paracancerous tissue samples were collected at the Department of Urology, Zhongnan Hospital of Wuhan University (approval number: 2 021 125), and informed consent was obtained from all individuals. Postoperative pathology examination confirmed their histological nature. BLCA tissue microarrays (TMA) with corresponding clinical data (HBlaU079Su01, containing 63 tumor tissues and 16 paracancerous tissues) were supplied by Shanghai Outdo Biotech Co., Ltd. (http://www.superchip.com.cn/index.html).

### Cell Culture and Transfections

All cell lines (SV‐HUC‐1, SCaBER, 5637, J82, T24, RT4, UM‐UC‐3, and HEK293T) were obtained from the Cell Bank of the Chinese Academy of Science (Shanghai, China). MEM supplemented with 10% fetal bovine serum was used to maintain the UM‐UC‐3 cells. DMEM containing 10% fetal bovine serum was used to maintain the HEK293T cells. RPMI 1640 medium supplemented with 10% fetal bovine serum was used to maintain SV‐HUC‐1, SCaBER, 5637, J82, T24, and RT4 cells. All cell lines passed cell authentication and were not contaminated by mycoplasma. The plasmids and siRNAs were transfected using Lipofectamine 3000 (L3000015, Invitrogen). For the plasmids, transfections were performed when cells were cultured to be 70–90% confluent in 6‐well plates. Diluted Lipofectamine 3000 reagent with 100 µL Opti‐MEM medium in tube 1. Diluted the plasmids with 100 µL Opti‐MEM medium and added P3000 reagent in tube 2. Added diluted plasmids to each tube of diluted Lipofectamine 3000 reagent, mixed well, and incubated for 15 mins at room temperature (RT). Added DNA‐lipid complex to cells and incubated cells for 2–3 days at 37 °C. To transfect cells with the siRNAs, followed the protocol as described for the plasmids but do not add P3000 reagent when diluting the siRNAs.

### RNA Sequencing

T24 cells were transfected with *siNC* and *siUSP52* for 2 days. Total RNA was extracted using the TRIzol reagent (Invitrogen, USA). The purity and quantification of RNA were evaluated with the NanoDrop One instrument (Thermo Scientific, USA). RNA integrity was assessed using the Agilent 2100 Bioanalyzer (Agilent Technologies, USA). Then the libraries were constructed using VAHTS Universal V6 RNA‐seq Library Prep Kit according to the manufacturer's instructions. Transcriptome sequencing and analysis were conducted by OE Biotech Co., Ltd. (Shanghai, China). Differential expression analysis was performed with the R package “DEseq2”. A *q*‐value < 0.05 and a fold change > 1.5 were set as the thresholds for significantly DEGs.

### Gas Chromatography‐Mass Spectrometry (GC‐MS) Untargeted Metabolomic Analysis

T24 cells were transfected with *siNC* and *siUSP52* for 2 days and stored all the samples at −80 °C. GC‐MS untargeted metabolomics and data reanalysis were executed by Luming Biotech Co., Ltd. (Shanghai, China). Differentially abundant metabolites with *p*‐value < 0.05 were selected. KEGG pathway enrichment was performed using the “clusterProfiler” package in R software (version 4.1.2).

### Deubiquitinase siRNA Library Screening

A human ON‐TARGETplus Deubiquitinating Enzyme siRNA library (G‐104705, Dharmacon) targeting 96 members of the deubiquitinating enzyme family was obtained from Thermo Fisher Dharmacon. The ON‐TARGETplus siRNA designs and modifications reduced off‐target effects while maintaining high silencing potency for high‐confidence screening results. To identify deubiquitinases involved in regulating the protein level of xCT, HEK293T cells were transfected with negative control siRNA or 25 nm DUBs siRNAs for 60 h in the 12‐well plates. The cells were collected for total protein extraction and subsequent Western blot analysis.

For the second‐round screen, T24 cells were transfected with negative control siRNA or 25 nm top 10 DUBs siRNAs for 24 h in the 96‐well plates and treated with or without 2 µm Erastin for 48 h. Cell viability was measured with an MTT solution using a SpectraMax M2 multi‐mode microplate reader (Molecular Devices, USA).

### Animal Experiments

To generate stable *USP52* knockdown cells (shUSP52) or negative control (shNC) cells, the lentiviruses LV‐shUSP52 and LV‐shNC were obtained from GenePharma (Shanghai, China), and puromycin (540 222, Sigma) was used to screen the lentivirus‐infected cells.

Four‐week‐old male BALB/c nude mice purchased from Gempharmatech Co., Ltd. (Jiangsu, China) were housed in the Animal Experimental Center of Zhongnan Hospital of Wuhan University. After adaptation for one week, the mice were randomly divided into two groups, each containing 14 mice, and a total of 5 × 10^6^ shNC and shUSP52 T24 cells resuspended in 100 µL of PBS were subcutaneously injected into the left posterior flanks. The progression of subcutaneous tumors was monitored by bidimensional measurements with a vernier caliper every three days until the endpoint. The modified ellipsoidal formula *v* = length × width^2^ × 1/2 was used for calculating the tumor size. Nine days after tumor cell injection, a two‐week course of intraperitoneal injections of 40 mg kg^−1^ IKE (S8877, Selleck) or an equal volume of dimethyl sulfoxide (DMSO, D103277, Aladdin) was administered to the mice every other day. Upon completion of the drug treatment, all mice were sacrificed and tumor samples were collected for weighing, imaging, and subsequent histological examination. Experiments on animals were approved by the Experimental Animal Welfare and Ethics Committee of Zhongnan Hospital (approval number: ZN2023184).

### siRNA, shRNA, and Plasmids

*siUSP52s* and *shUSP52* were synthesized by GenePharma (Shanghai, China), whose detailed information are listed in Tables S3 and S4 (Supporting Information).

The human xCT‐Flag‐tagged and USP52‐Myc‐tagged plasmids were purchased from Miaoling Biology Co., Ltd. (Wuhan, China). The human xCT‐ΔNTD‐Flag‐tagged (deletion xCT‐N‐terminal domain mutant), xCT‐ΔCTD‐Flag‐tagged (deletion xCT C‐terminal domain mutant), xCT‐Flag‐tagged (K4R, K12R, K30R, K37R, K222R, K483R, K4R/K12R), USP52‐WD40‐Myc‐tagged (amino acids 1–499), USP52‐UCH‐Myc‐tagged (amino acids 499–949), USP52‐EXO‐Myc‐tagged (amino acids 949–1203), USP52‐ΔUCH‐Myc‐tagged (deletion UCH domain mutant) and USP52‐C528A/C530A‐Myc‐tagged plasmids were constructed by standard subcloning in our laboratory. The HA‐K6, HA‐K11, HA‐K27, HA‐K29, HA‐K33, HA‐K48, HA‐K63, and HA‐WT Ub plasmids were kindly provided as a gift from Prof. Guoliang Qing at Wuhan University, China.

### RNA Isolation and Quantitative Reverse Transcription PCR (qRT‐PCR)

These manipulations were performed following our previous works.^[^ ^51^ ^]^ Briefly, total RNA was extracted with HiPure Total RNA Mini Kit (R4111‐03, Magen, China). The quality and concentration of RNA were evaluated with the NanoDrop One instrument. A ReverTra Ace qPCR RT Kit (FSQ‐101, TOYOBO, Japan) was used to reverse‐transcription RNA to cDNA. The qRT‐PCR was performed using SYBR Green Supermix (Bio‐Rad, China) with the StepOnePlus Real‐Time PCR System (Thermo Fisher, USA). Table S5 (Supporting Information) lists the primers used in this study.

### Antibody and Compounds

A list of the antibodies and compounds is included in Tables S6 and S7 (Supporting Information), respectively.

### Western Blot and Immunoprecipitation (IP) Analysis

Western blot experiments were carried out as previously described.^[^ ^52^ ^]^ Cells were lysed with a mixture containing RIPA buffer, 1 mm PMSF, and phosphatase inhibitors on ice for 30 mins and the cell supernatants were collected by centrifugation. Then, boiled the whole cell lysates with 1× SDS‐PAGE loading buffer at 100 °C for 10 mins. The total proteins were separated by SDS‐PAGE gel, followed by transfer to a polyvinylidene difluoride (PVDF) membrane and blocked by 5% skim milk in TBST buffer (10 mm Tris‐HCI, 150 mm NaCl, and 2% Tween 20). The primary antibodies were incubated with PVDF membrane at 4 °C overnight. Then, washed the membranes, added and incubated with the secondary antibodies in 2% skim milk at RT for 1 h. Finally, the membranes were exposed with an Omni‐ECL Light Chemiluminescence Kit (Epizyme Biotech, SQ202L, China) by a chemiluminescence and gel imager (BioSpectrum 515 lmaging System, UVP).

Co‐IP assays were performed by a BeaverBeads Protein A/G Immunoprecipitation Kit (22202‐100, Beaver). Briefly, cells were collected (endogenous IP: 2 × 10^7^ T24 or 5637; exogenous IP: 1 × 10^6^ HEK293T cells) and lysed with a mixture containing IP binding buffer, 1 mm PMSF, and phosphatase inhibitors on ice for 30 mins. Then, the cell supernatants were collected into a 1.5 mL EP tube by centrifugation and incubated with 5–10 µg mL^−1^ antibody using a rotary mixer at 4 °C overnight. Magnetic beads (20 µL) were placed into an EP tube to generate a magnetic beads‐antibody‐antigen complex suspension. Magnetic separation was performed after 1 h of incubation, followed by gentle washing of the magnetic beads three times. Finally, boiled the magnetic beads with 1× SDS‐PAGE loading buffer at 100 °C for 10 mins for subsequent Western blot analysis.

### Cell Viability Assay

As described previously,^[^ ^53^ ^]^ cells were transfected in the 6‐well plates for 48 h and 2000 cells per well were seeded in the 96‐well plates. Then, added 20 µL well^−1^ of MTT (methyl thiazolyl tetrazolium, Sigma) solution, incubated the plates for 4 h, added 200 µL well^−1^ of DMSO, and dissolved the solution for 10 mins in turn. Finally, measured the absorbance using a SpectraMax M2 multi‐mode microplate reader for five consecutive days.

For drug treatment, 5000 transfected cells were seeded in the 96‐well plates for 24 h and treated with different drugs as indicated for 48 h. Cell viability was measured in the above manner.

### Colony Formation Assay

About 1000 transfected cells per well were seeded in the 6‐well plates and cultured for 9–12 days. Next, cells were fixed with 4% paraformaldehyde, stained with 0.1% crystal violet dye, and imaged with a camera.

### Cell Death Assay

Cells were seeded and transfected for 24 h, followed by treat with appropriate drugs for 48 h in the 6‐well plates. Collected and pelleted the cells by centrifugation, discarded the supernatant, and washed the cells with PBS. Then, the cell samples were incubated with 3 µm Propidium Iodide (PI, Invitrogen) in 1 mL PBS at RT for 15 mins. Dead cells (PI‐positive cells) were analyzed using a flow cytometer (CytoFlex, Beckman). An example gating strategy for flow cytometry analysis of PI‐positive cells was showed in Figure S9A (Supporting Information).

### Cell Apoptosis Analysis

An Annexin V‐FITC/PI Apoptosis Detection Kit (Simu Biotech, China) was used to detect apoptotic cell rates. Cells were seeded and transfected for 48 h in the 6‐well plates. Collected and pelleted the cells by centrifugation, discarded the supernatant, and washed the cells with PBS. Then, the cell samples were incubated with a mixture containing 500 µL Binding buffer, 5 µL Annexin V‐FITC, and 5 µL PI Solution away from light at RT for 15 mins. Apoptotic cells were analyzed by a flow cytometer. Example gating strategy for flow cytometry analysis of apoptotic cells was showed in Figure S9B (Supporting Information).

### Lipid ROS Analysis

Cells were seeded and transfected for 24 h, followed by treat with appropriate drugs for 24 h in the 6‐well plates. A Lipid Peroxidation Probe BODIPY‐C11 581/591 (L267, Dojindo) stock solution was diluted and added to the plates. After incubation at 37 °C for half an hour, washed the cells with PBS, and evaluated the level of fluorescence with a flow cytometer. Oxidation of BODIPY‐C11 resulted in a shift of the fluorescence emission peak from 590 to 510 nm proportional to lipid ROS generation. The FL1 channel signal in live cells was plotted as shown in the Figures. The gates or called threshold to define what constituted an increased FITC/phycoerythrin fluorescence ratio were set based on untreated cancer cells, a condition that represents cells with little or no lipid peroxidation. The lipid ROS levels were analyzed with FlowJo software (version 10.8). An example gating strategy for flow cytometry analysis of BODIPY‐C11 581/591 oxidation in cells was showed in Figure S9C (Supporting Information).

### Glutathione (GSH) Assay

After transfected for 48 h, cells were trypsinized and seeded in the 96‐well plates. The level of GSH in cells was measured using a GSH‐Glo Glutathione Assay Kit (V6911, Promega). Discarded the culture medium, added 100 µL well^−1^ of 1× GSH‐Glo reagent, and incubated at RT for 30 mins. Then, added 100 µL well^−1^ of reconstituted luciferin detection reagent and incubated at RT for 15 mins. A microplate reader was used to obtain luminescence values, and the GSH levels were calculated according to a prepared GSH standard curve.

### Cell Ferrous Iron Assay

The level of ferrous iron in cells was measured using a Cell Ferrous Iron Colorimetric Assay Kit (E‐BC‐K881‐M, Elabscience). After transfected for 48 h in the 6‐well plates, 1 × 10^6^ cells were trypsinized and collected, followed by lyse with 200 µL Reagent 1 on ice for 10 mins. Then, collected the supernatant by centrifugation. Mixed 80 µL samples with 80 µL Reagent 2 as a control group and mixed 80 µL samples with 80 µL Reagent 3 as an experimental group in the 96‐well plates. After incubation at 37 °C for 10 mins, a microplate reader was used to obtain luminescence values, and the ferrous iron levels were calculated by a standard curve and a prepared formula according to the product manual.

### EdU Staining Assay

A BeyoClick EdU Cell Proliferation Kit (C0071S, Beyotime) was used to perform the EdU staining assay. Cells were cultured to a relatively high density and treated with 10 µm EdU at 37 °C for 2 h. Next, the cell sections were fixed, permeabilized, and washed. Then, added 500 µL well^−1^ of click additive solution and incubated the plates for 30 mins in the dark. For nuclear staining, cell sections were incubated with 1 mL DAPI for 10 mins. Finally, cell sections were photographed with a confocal laser microscope (C2^+^, Nikon, Japan), and the proportion of EdU‐positive cells was calculated with ImageJ software (version 1.54).

### Light Microscopy and TEM

For light microscopy, cells were seeded and transfected for 24 h, followed by treat with appropriate drugs for 48 h in the 6‐well plates. Phase contrast images were obtained using a light microscope (IX73, Olympus) equipped with Image‐Pro Plus software (version 6.0). For TEM, cells were trypsinized and collected by centrifugation, followed by a fix with 2.5% glutaraldehyde solution away from light at RT for 30 mins. Then, cell sections were prepared followed our previous methods,^[^ ^54^ ^]^ and the ultrastructure of the mitochondria was captured by a TEM (HT7700, Hitachi).

### Immunofluorescence

We cultured the cells in a plate covered with a cover glass. Then, the cell sections were fixed with 4% paraformaldehyde for 20 mins, permeabilized with 0.1% Triton X‐100 in PBS buffer for 5 mins, blocked with 1% BSA in PBS buffer for 1 h, incubated with primary antibodies in 1% BSA overnight, incubated with secondary antibodies in 1% BSA away from light for 1 h, stained with 0.5 µg mL^−1^ DAPI away from light for 10 mins and immobilized on a microslide. A confocal laser microscope (C2^+^, Nikon, Japan) was used to capture the images.

### Histology and Immunohistochemistry (IHC)

For hematoxylin and eosin staining, tissue sections were deparaffinized, hydrated, stained with 10% hematoxylin and 1% eosin, and dehydrated. For IHC staining, paraffin‐embedded tissue sections were sequentially deparaffinized, hydrated, blocked, incubated with primary and secondary antibodies, and visualized with a DAB color solution. The stained sections were photographed by a multifunction scanning microscope (Aperio VERSA 8, Leica). The average optical density of proteins was assessed with ImageJ software (version 1.54).

### Protein Stability Assay

Cells were transfected with the corresponding siRNAs or plasmids for 48 h. After treated with 50 µg mL^−1^ CHX as indicated hours, the cells were collected for protein extraction and subsequent Western blot assay. The relative protein level of xCT was calculated using ImageJ software (version 1.54) and normalized to that of the loading control.

### Protein Ubiquitination Assay

Cells were transfected with the corresponding siRNA or plasmids for 48 h. After incubated with 10 µm MG132 for 6 h, the cells were lysed on ice for 30 mins using a mixture containing RIPA lysis buffer, 1 mm PMSF, and phosphatase inhibitors. Then, the cell supernatants were incubated overnight with the corresponding antibody and then subjected to magnetic bead incubation for 1 h. Finally, polyubiquitinated xCT was examined via Western blot analysis.

### Statistical Analysis

All statistical analyses were conducted through GraphPad Prism (version 9.0) with two‐tailed Student's *t‐*test, one‐way or two‐way ANOVA followed by Tukey's multiple comparison test, depending on the situation. Survival analysis was carried out using the Kaplan–Meier method. The data were shown as the mean ± standard deviation (S.D.). *p* < 0.05 indicated a significant difference and *p*‐value numbers were shown for each statistical comparison result. Detailed statistical methods were described in the figure legends or accompanying text. The number (*n*) of tested samples was shown in the figures or legends, where applicable.

### Data Availability

The RNA‐seq data generated in this study were deposited in the GEO database under accession code GSE263111. The publicly available TCGA‐BLCA cohort data (comprising 408 tumor samples and 19 normal samples) were obtained from the GDC Data Portal website (https://portal.gdc.cancer.gov/). The remaining data are available within the Article, Supporting Information, or Original Data file. Source data are provided as a Source Data file. Source data are provided with this paper. The original code used during the study was provided at Zenodo: https://zenodo.org/records/10972307.

## Conflict of Interest

The authors declare no conflict of interest.

## Author Contributions

J.L., Y.L., S.C., and G.W. contributed equally to this work. K.Q. and X.W. designed and supervised the study. J.L., Y.L., S.C., and G.W. performed the most experiments. J.L., G.W., W.J., W.J., M.L., Y.W., J.Y., H.W., R.Z., F.Z., L.J., Y.Z., and Y.X. analyzed the results and collected the specimens. J.L., Y.L., and L.J. wrote the first draft. K.Q. and X.W. critically revised drafts of the manuscript. All authors reviewed the manuscript.

## Supporting information



Supporting Information

Supplemental Table 1

## Data Availability

The data that support the findings of this study are openly available in Gene Expression Omnibus at https://www.ncbi.nlm.nih.gov/geo/, reference number GSE263111.
