# Supplementary material for: Deubiquitylase USP52 Promotes Bladder Cancer Progression by Modulating Ferroptosis through Stabilizing SLC7A11/xCT
Source: Adv Sci (Weinh). 2024 Oct 11;11(45):2403995. doi: 10.1002/advs.202403995 (PMC11615784; doi:10.1002/advs.202403995)
Supplement: Supplementary file 1 — Supporting Information [file ADVS-11-2403995-s002.pdf]

ADVANCED SCIENCE
Open Access

Supporting Information

for *Adv. Sci.*, DOI 10.1002/advs.202403995

Deubiquitylase USP52 Promotes Bladder Cancer Progression by Modulating Ferroptosis through Stabilizing SLC7A11/xCT

Jianmin Liu, Yongwen Luo, Siming Chen, Gang Wang, Wan Jin, Wenyu Jiang, Mingxing Li, Yejinpeng Wang, Jingtian Yu, Houyi Wei, Renjie Zhang, Fenfang Zhou, Lingao Ju, Yi Zhang, Yu Xiao, Kaiyu Qian and Xinghuan Wang**

Supplementary Information

Deubiquitylase USP52 Promotes Bladder Cancer Progression by Modulating Ferroptosis through Stabilizing SLC7A11/xCT

Figure S1-S9: Pages 2-14

Table S1-S7: Pages 15-21

Figures S1-S9

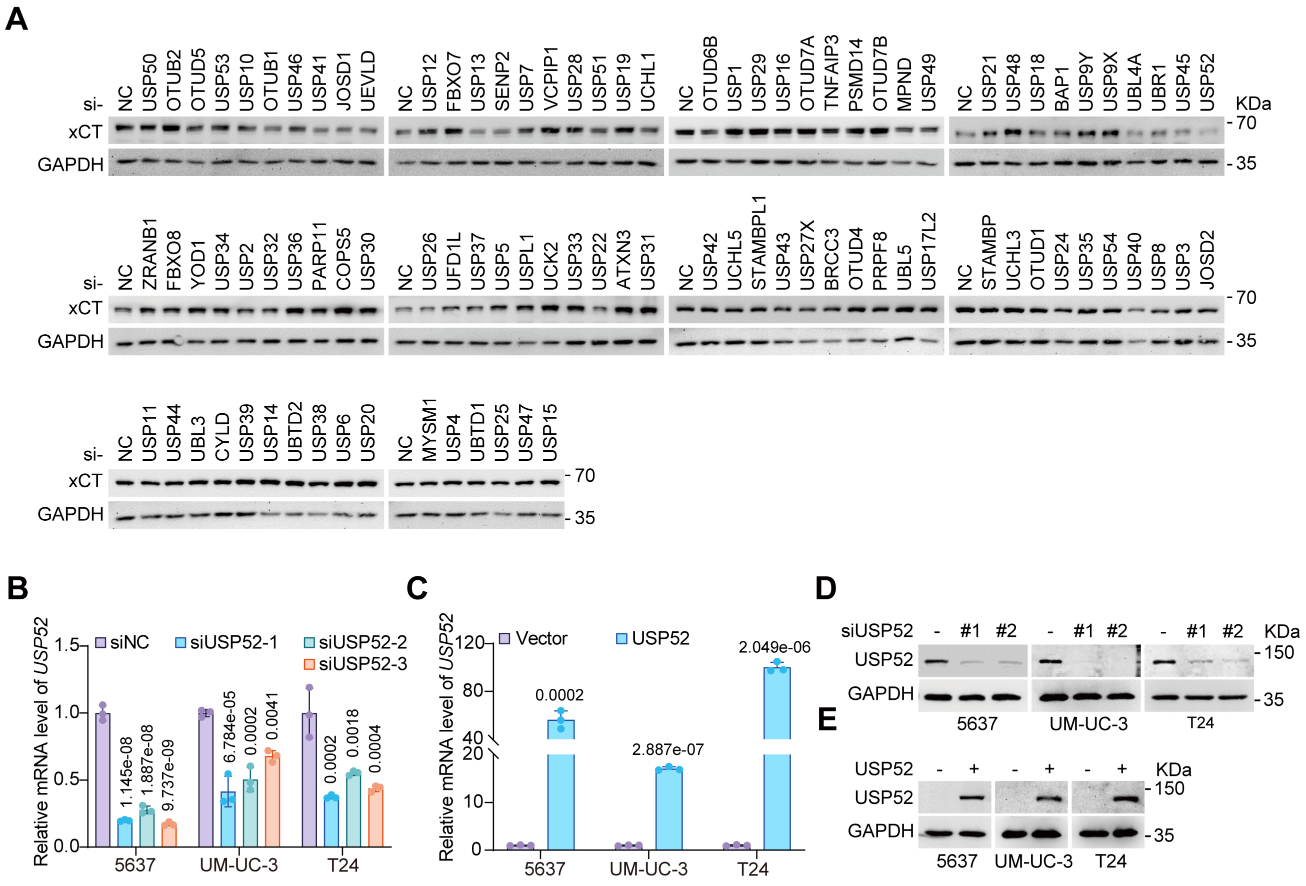

Figure S1. Validation of knockdown or overexpression efficiency. Related to Figure 1.

(A) A DUB siRNA library specific to 96 DUBs or negative control siRNA was transfected into HEK293T cells for 3 days in the 12-well plates. The cells were collected for Western blot assay and the protein expression of xCT was showed with GAPDH as a loading control. **(B, D)** BLCA cells were transfected with indicated siRNAs (*siNC* and *siUSP52s*) for 48 hrs, followed by collection for analysis. Validation of knockdown efficiency of three *USP52*-specific siRNA by qRT-PCR assays (B) and Western blot assays (D) in 5637, UM-UC-3 and T24 cells. **(C, E)** BLCA cells were transfected with vector or Myc-USP52 for 48 hrs, followed by collection for analysis. Validation of Myc-USP52 overexpression efficiency in 5637, UM-UC-3 and T24 cells by qPCR assays (C) and Western blot assays (E). Data represent mean ± SDs, with n = 3 (B, C) independent repeats. Statistical significance was determined by two-tailed unpaired Student's t-test (B, C).

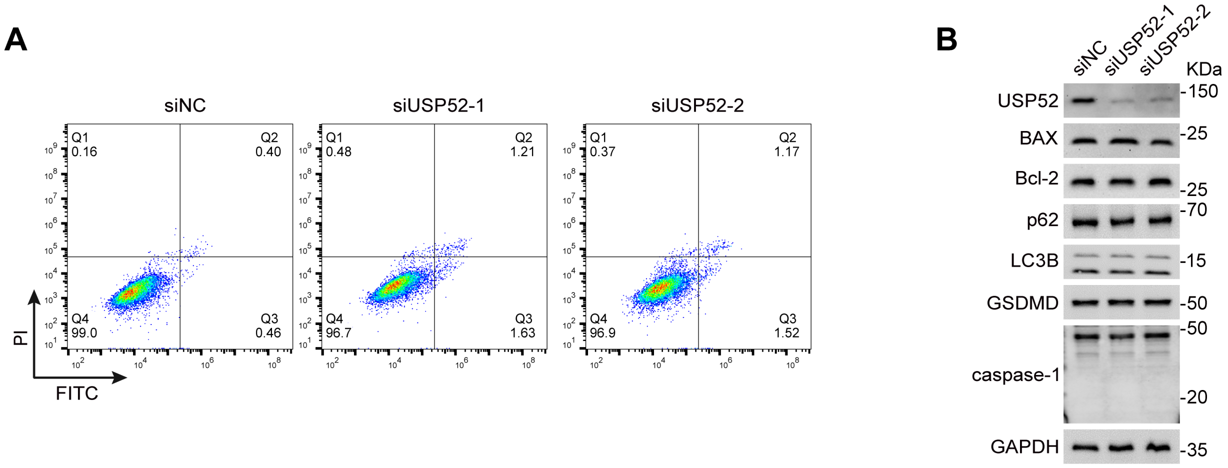

Figure S2. ***USP52*** **depletion has no effect on cell apoptosis, autophagy and pyroptosis in bladder cancer cells. Related to Figure 2.**

(A) T24 cells were transfected with indicated siRNAs (*siNC* and *siUSP52s*) for 48 hrs, followed by collection for Annexin V-FITC/PI staining. Apoptotic cells were analysed by a flow cytometer. **(B)** T24 cells were transfected with indicated siRNAs (*siNC* and *siUSP52s*) for 48 hrs, followed by collection for Western blot assay. Alteration of proteins involved in cell apoptosis, autophagy and pyroptosis were showed.

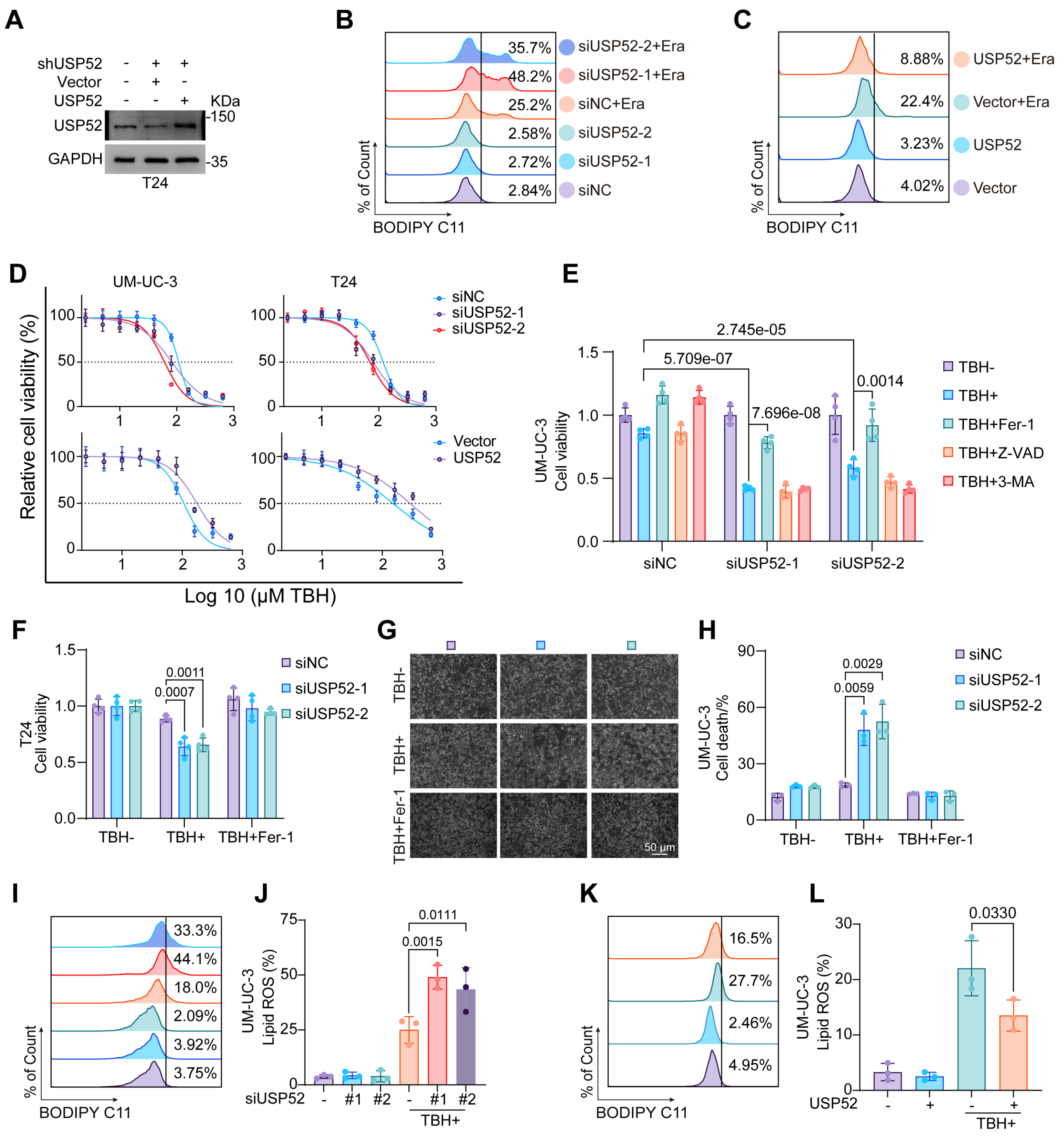

Figure S3. *USP52* depletion promotes ferroptosis in bladder cancer cells. Related to Figure 2.

(A) *USP52*-depleted T24 cells were transfected with vector or USP52 plasmids for 48 hrs, followed by collection for Western blot assay. The protein expression of USP52 in *USP52*-depleted cells with or without USP52 re-expression were showed. **(B, C)** T24 cells were transfected with indicated siRNAs or plasmids for 24 hrs and treated with 2 μM Era for 24 hrs. Then, collected and stained cells with BODIPY-C11 probe. Representative data of lipid peroxidation assessed by a flow cytometry after BODIPY-C11 staining in *USP52*-knockdown (B) or USP52-overexpressed (C) cells were showed. **(D)** BLCA cells were transfected with siRNAs or plasmids for 24 hrs. Then, 5000 transfected cells per well were seeded in the 96-well plates for 24 hrs and treated with different concentrations of tert-butyl hydroperoxide (TBH) for 48 hrs. Cell viability of

USP52-knockdown (row 1) and USP52-overexpressed cells (row 2) following treatment with different concentrations of TBH were measured via MTT assay. **(E)** UM-UC-3 cells were transfected with indicated siRNAs (*siNC* or *siUSP52s*) for 24 hrs. Then, 5000 transfected cells per well were seeded in the 96-well plates for 24 hrs and treated with 40 μM TBH alone or in combination with 2 μM Fer-1, 5 μM Z-VAD, or 5 mM 3-MA for 48 hrs. Cell viability of indicated groups were measured via MTT assay, and the absorbance was normalized to the TBH- group. **(F)** T24 cells were transfected with indicated siRNAs (*siNC* or *siUSP52s*) for 24 hrs. Then, 5000 transfected cells per well were seeded in the 96-well plates for 24 hrs and treated with 40 μM TBH alone or in combination with 2 μM Fer-1 for 48 hrs. Cell viability of indicated groups were measured via MTT assay, and the absorbance was normalized to the TBH- group. **(G)** Representative phase-contrast images of *USP52*-knockdown cells treated with 40 μM TBH alone or in combination with 2 μM Fer-1 for 48 hrs. Scale bar: 50 μm. **(H)** UM-UC-3 cells were transfected with indicated siRNAs (*siNC* or *siUSP52s*) for 24 hrs and treated with 40 μM TBH alone or in combination with 2 μM Fer-1 for 48 hrs. Then, collected and stained cells with pyridine iodide (PI) staining solution. Cell death rates (PI-positive cells) were calculated via a flow cytometer. **(I, J)** UM-UC-3 cells were transfected with indicated siRNAs (*siNC* or *siUSP52s*) for 24 hrs and treated with 40 μM TBH for 24 hrs. Then, collected and stained cells with BODIPY-C11 probe. Lipid peroxidation was assessed by a flow cytometry. **(K, L)** UM-UC-3 cells were transfected with vector or USP52 plasmids for 24 hrs and treated with 40 μM TBH for 24 hrs. Then, collected and stained cells with BODIPY-C11 probe. Lipid peroxidation was assessed by flow cytometry. Data represent mean ± SDs, with n = 3 (H, J, L) or 4 (D-F) independent repeats. Statistical significance was determined by one-way ANOVA with Tukey's multiple comparisons (E, F, H, J, L).

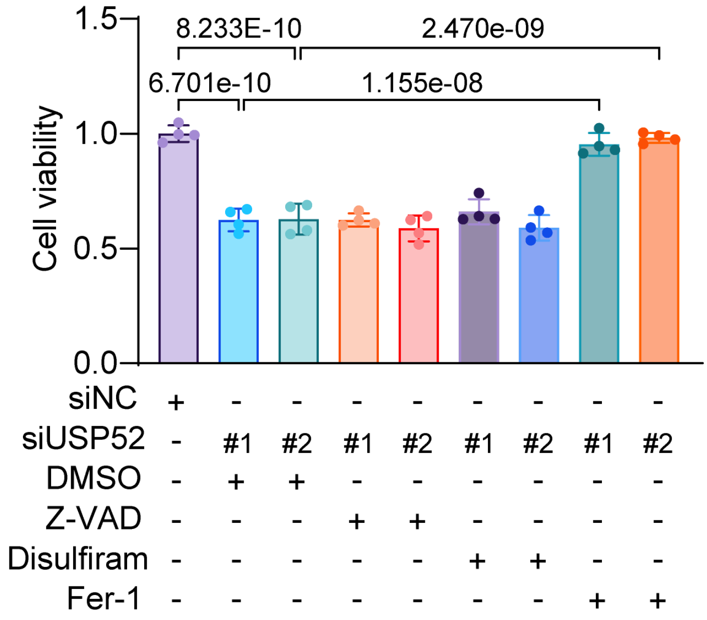

Figure S4. Depletion of *USP52* inhibits bladder cancer cell growth in a ferroptosis-dependent manner. Related to Figure 3.

T24 cells were transfected with indicated siRNAs (*siNC* or *siUSP52s*) for 24 hrs. Then, 5000 transfected cells per well were seeded in the 96-well plates for 24 hrs and treated with DMSO, 5 μM Z-VAD, 50 μM Disulfirm or 2 μM Fer-1 for 48 hrs. Cell viability of indicated groups were measured via MTT assay, and the absorbance was normalized to the *siNC* group. Data represent mean ± SDs, with n = 4 independent repeats. Statistical significance was determined by one-way ANOVA with Tukey's multiple comparisons.

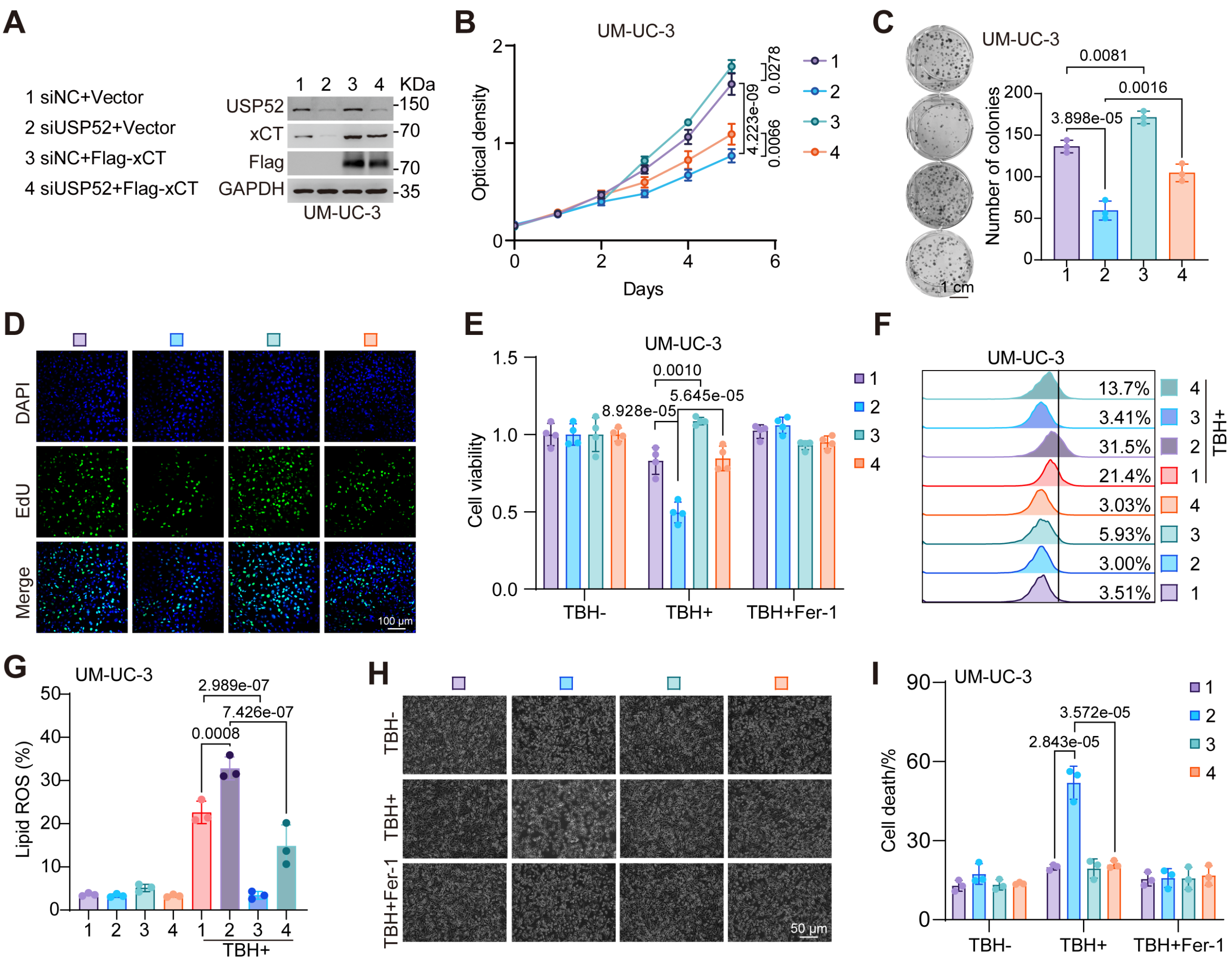

Figure S5. Depletion of *USP52* inhibits bladder cancer progression through decreasing xCT and promoting ferroptosis. Related to Figure 4.

(A) UM-UC-3 cells were transfected with indicated siRNAs (*siNC* and *siUSP52*) for 24 hrs, followed by transfection with vector of Flag-xCT plasmids for 48 hrs. Four groups in this figure were indicated as 1, 2, 3 and 4. Cells were collected and lysed for total protein extraction. Western blot assays showing the expression of USP52 and xCT in *USP52*-silenced UM-UC-3 cells with or without xCT re-expression. **(B)** About 2000 transfected cells per well were seeded in the 96-well plates, followed by assessment of cell viability with MTT assay for five consecutive days. Cell proliferation curves of *USP52*-silenced UM-UC-3 cells with or without xCT re-expression were showed. **(C)** About 1000 transfected cells per well were seeded and cultured in the 6-well plates for 9-12 days, followed by fixation, staining and photograph. Representative images (left panel) and statistical graph (right panel) of colony formation assays of *USP52*-silenced T24 UM-UC-3 cells with or without xCT re-expression were showed. **(D)** Transfected UM-UC-3 cells were fixed and stained with EdU staining kit. Images of EdU-positive cells captured by a confocal microscope in *USP52*-silenced cells with or without xCT re-expression were showed. Blue indicated nuclear staining and green indicated EdU-positive cells. **(E)** About 5000 transfected UM-UC-3 cells per well were seeded in the 96-well plates for 24 hrs and

treated with 40 μM TBH alone or in combination with 2 μM Fer-1 for 48 hrs. Cell viability of indicated groups were measured via MTT assay, and the absorbance was normalized to the TBH- group. **(F, G)** Transfected UM-UC-3 cells were treated with 40 μM TBH for 24 hrs. Then, collected and stained cells with BODIPY-C11 probe. Lipid peroxidation was assessed by flow cytometry. Representative data (F) and statistical graph (G) of lipid ROS in UM-UC-3 cells with indicated groups were showed. **(H)** Representative phase-contrast images of transfected UM-UC-3 cells treated with 40 μM TBH alone or in combination with 2 μM Fer-1 for 48 hrs. **(I)** Transfected UM-UC-3 cells were treated with 40 μM TBH alone or in combination with 2 μM Fer-1 for 48 hrs. Then, collected and stained cells with pyridine iodide (PI) staining solution. Cell death rates (PI-positive cells) were calculated via flow cytometer. Scale bar: 1 cm (C), 100 μm (D) and 50 μm (H). Data represent mean ± SDs, with n = 3 (C, G, I) or 4 (E) or 5 (B) independent repeats. Statistical significance was determined by one-way ANOVA with Tukey's multiple comparisons (B, C, E, G, I).

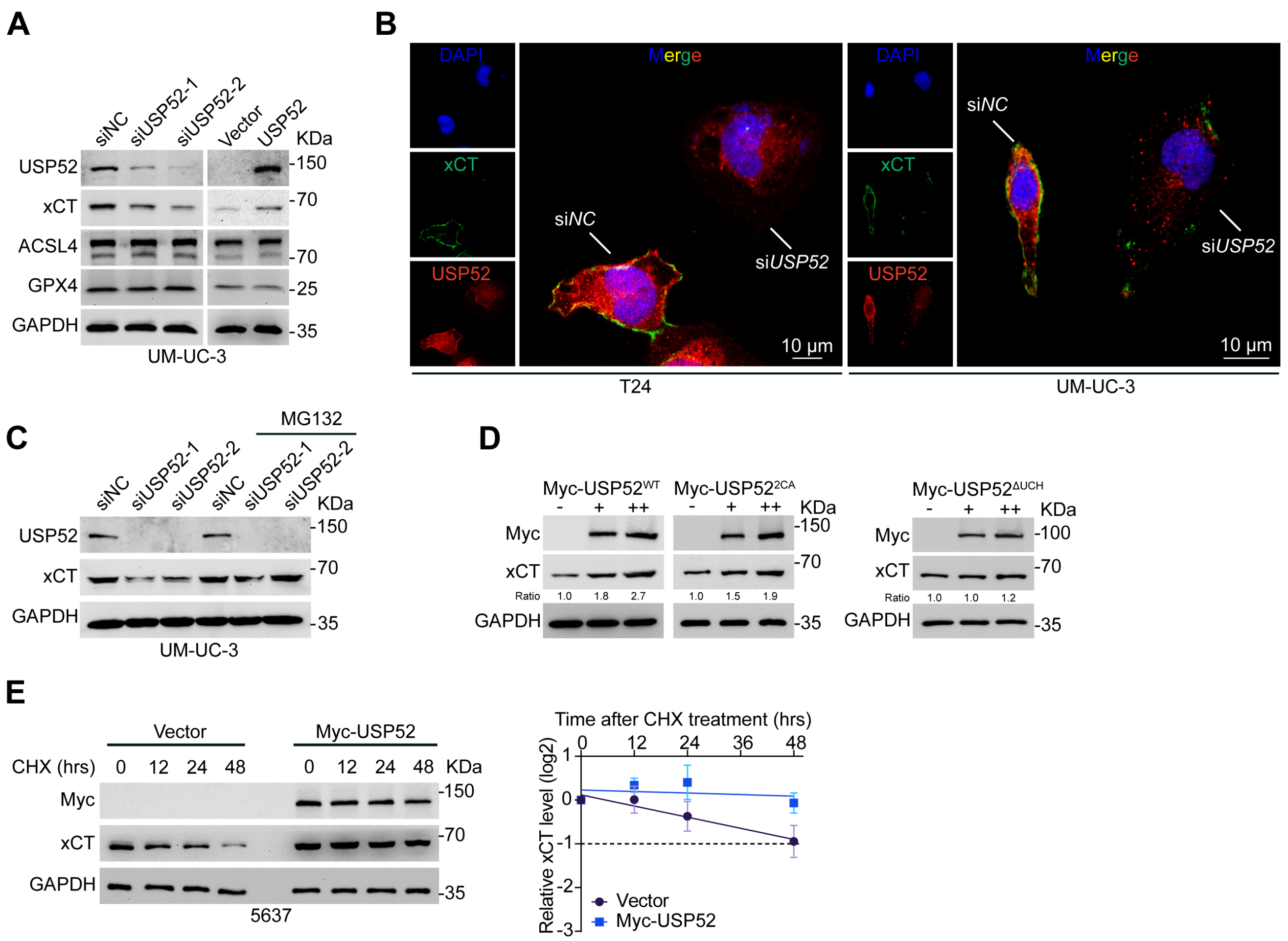

Figure S6. USP52 interacts with xCT and maintains its protein stability. Related to Figure 5.

(A) UM-UC-3 cells were transfected with indicated siRNAs or plasmids for 48 hrs, followed by collection for Western blot assay. The protein levels of ferroptosis-related proteins in UM-UC-3 cells with *USP52* knockdown or USP52 overexpression were showed**. (B)** BLCA cells were transfected with indicated siRNAs (*siNC* and *siUSP52*) for 48 hrs, followed by trypsinization and collection. After mixed *siNC* and *siUSP52* cells in equal proportions, cells were re-seeded in the 6-well plates covered with a cover glass for immunofluorescence assay. Images of immunofluorescence staining of USP52 (red) and xCT (green) in T24 and UM-UC-3 cells were captured by confocal microscopy. Cells with lower USP52 expression were considered as USP52-silenced cells. Scale bar: 10 μm. **(C)** UM-UC-3 cells were transfected with indicated siRNAs (*siNC* and *siUSP52s*) for 48 hrs, followed by treatment with 10 μM 6 hrs MG132 before collection. Western blot analysis of USP52 and xCT in *USP52*-silenced UM-UC-3 cells with or without 10 μM MG132 treatment were showed. **(D)** T24 cells transfected with empty control or increasing amount of Myc-USP52, Myc-USP52-2CA or Myc-USP52-ΔUCH plasmids for 48 hrs, followed by collection for Western blot assay. The protein levels of endogenous xCT were showed. **(E)** 5637 cells were transfected with vector or Myc-USP52 plasmids for 48 hrs and treated with 50 μg/mL CHX as indicated hours, followed by collection for Western blot assay. Representative Western blot images showing the effect of USP52 overexpression on xCT

degradation in 5637 cells (left panel) and a statistical diagram of the results of the protein half-life assays (right panel). Data represent mean ± SDs, with n = 3 (E) independent repeats.

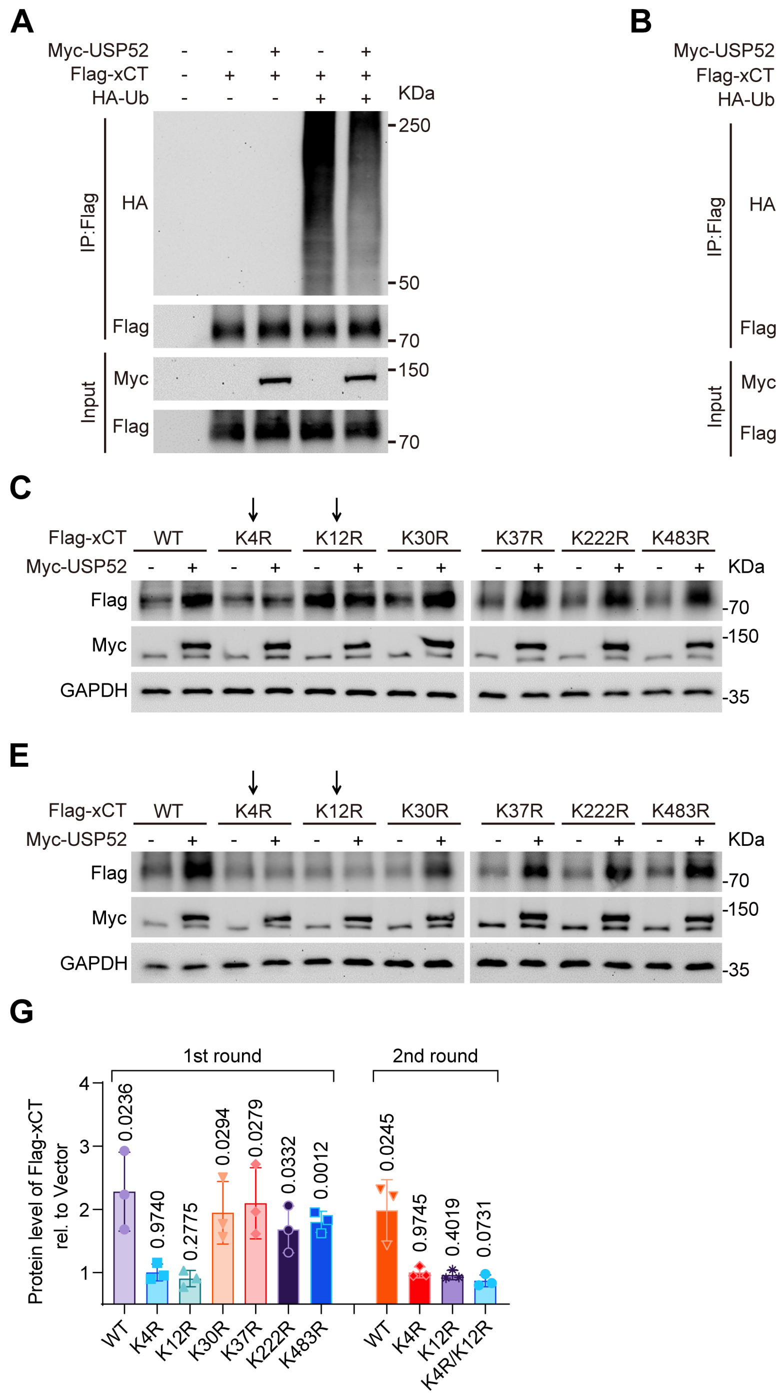

Figure S7. USP52 deubiquitinates xCT at K4 and K12. Related to Figure 6.

(A) HEK293T cells were transfected with the described plasmids as indicated for 48 hrs and then treated with 10 μM MG132 for 6 hrs. Cellular extracts were prepared for IP assays after Flag-IP followed by immunoblotting with anti-HA. **(B)** HEK293T cells were transfected with Flag-xCT and HA-Ub (K6O, K11O, K27O, K29O or K33O) together with empty control or Myc-USP52 as indicated for 48 hrs and then treated with 10 μM MG132 for 6 hrs. The IP assays were performed after Flag-IP followed by immunoblotting with anti-HA. **(C, E)** HEK293T cells were transfected with 1 μg wild-type Flag-xCT or six K-to-R mutants together with empty control or Myc-USP52 for 60 hrs in the first round of screening, followed by collection for Western

blot assay. The expression of Flag-xCT detected with an anti-Flag antibody were showed. **(D, F)** HEK293T cells were transfected with 1 μg wild-type Flag-xCT, K4R, K12R, or K4R/K12R mutants together with empty control or Myc-USP52 for 60 hrs in the second round of screening, followed by collection for Western blot assay. The expression of Flag-xCT detected with an anti-Flag antibody were showed. **(G)** The quantification of bands was analyzed by Image J and data are presented as mean values ± SDs from n = 3 independent experiments. Statistical significance was determined by two-tailed unpaired Student's t-test (G).

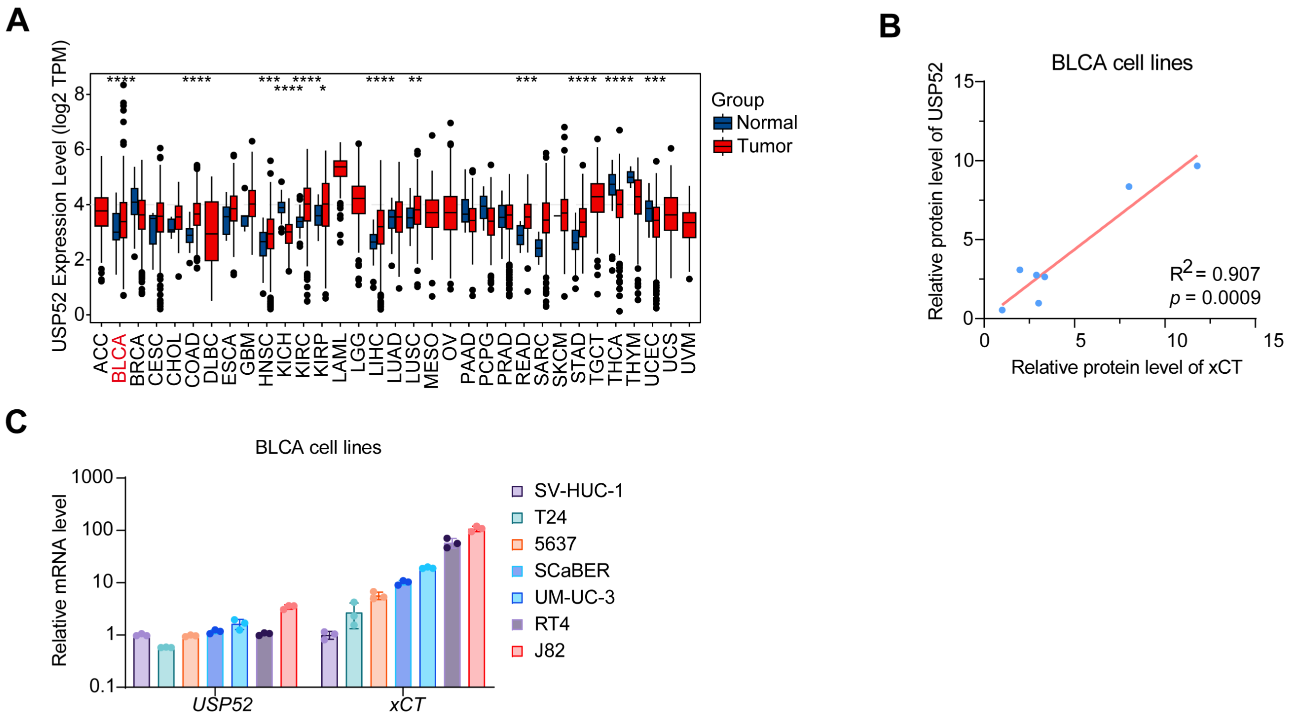

Figure S8. USP52 is positively correlated with xCT and is associated with BLCA progression and prognosis. Related to Figure 8.

(A) The mRNA expression distribution of *USP52* in pan-cancer from the TCGA database (*https://portal.gdc.cancer.gov/*), with the X-axis showing tumor names including BLCA, and the Y-axis showing *USP52* expression (log2 TPM). Red indicates *USP52* distribution in tumor tissue and blue represents normal tissue. **(B)** Western blot analyses of USP52 and xCT across multiple cell lines. Band intensity was quantified by densitometry with ImageJ software (GAPDH as a normalizer). The correlation coefficient and *p*-value are shown. **(C)** One normal uroepithelial cell line (SV-HUC-1) and six BLCA cell lines (T24, 5637, SCaBER, UM-UC-3, RT4, and J82) were analyzed by Western blot. The mRNA expression of *USP52* and *xCT* in these cell lines were normalized to SV-HUC-1. Abbreviations in (A) include: ACC: Adrenocortical carcinoma; BLCA: Bladder urothelial carcinoma; BRCA: Breast invasive carcinoma; CESC: Cervical squamous cell carcinoma and endocervical adenocarcinoma; CHOL: Cholangiocarcinoma; COAD: Colon adenocarcinoma; DLBC: Diffuse large B-cell lymphoma; ESCA: Esophageal carcinoma; GBM: Glioblastoma multiforme; HNSC: Head and neck squamous cell carcinoma; KICH: Kidney chromophobe; KIRC: Kidney renal clear cell carcinoma; KIRP: Kidney renal papillary cell carcinoma; LAML: Acute myeloid leukemia; LGG: Brain lower grade glioma; LIHC: Liver hepatocellular carcinoma; LUAD: Lung adenocarcinoma; LUSC: Lung squamous cell carcinoma; MESO: Mesothelioma; OV: Ovarian cancer; PAAD: Pancreatic adenocarcinoma; PCPG: Pheochromocytoma and paraganglioma; PRAD: Prostate adenocarcinoma; READ: Rectum adenocarcinoma; SARC: Sarcoma; SKCM: Skin cutaneous melanoma; STAD: Stomach adenocarcinoma; TGCT: Testicular germ cell tumors; THCA: Thyroid carcinoma; THYM: Thymoma; UCEC: Uterine corpus endometrial carcinoma. UCS: Uterine carcinosarcoma; UVM: Uveal melanoma; TPM: Transcripts per kilobase million. Statistical significance was determined by Pearson’s correlation (B).

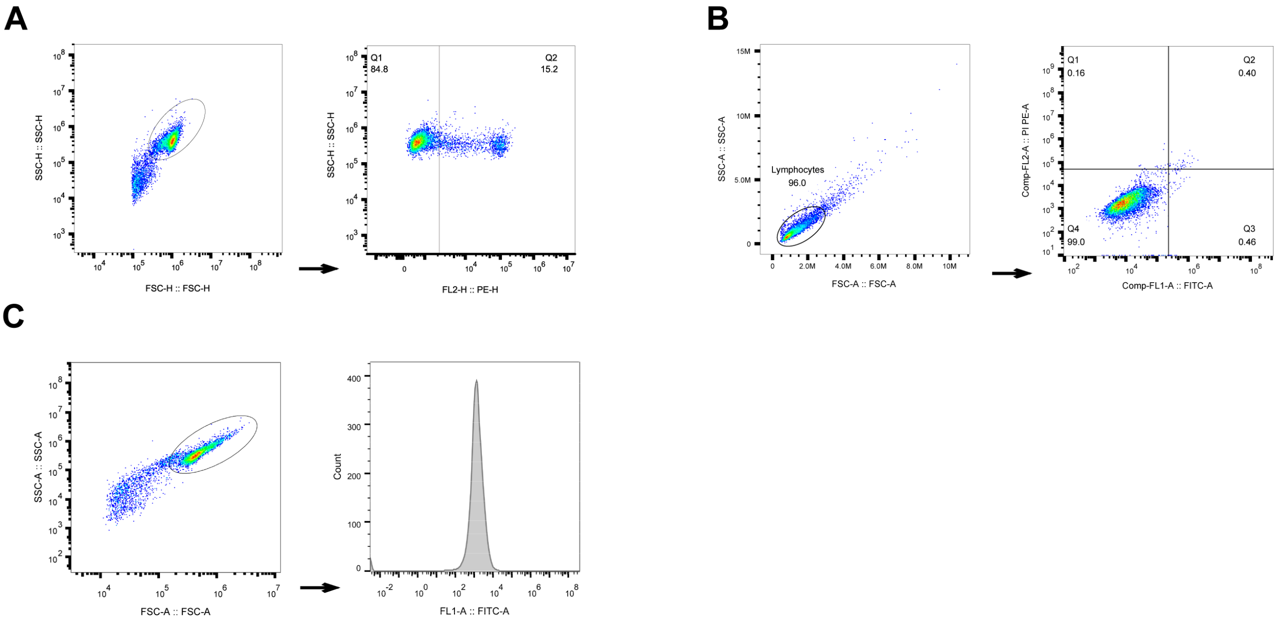

Figure S9. Gating strategy for flow analysis. Related to all figures of flow cytometry analysis in the manuscript.

(A) Example gating strategy for flow cytometry analysis of PI-positive cells. **(B)** Example gating strategy for flow cytometry analysis of apoptotic cells. **(C)** Example gating strategy for flow cytometry analysis of BODIPY-C11 581/591 oxidation in cells.

Tables S1-S7

Table S1. Clinicopathological characteristics of the Zhongnan Hospital cohort.

Patient ID	Gender	Age / ys	TNM Stage	Tumor Size / cm^3
1	Male	64	pT1NxMx	5*4*4
2	Male	89	pT1NxMx	7*3*1.5
3	Male	60	pTaNxMx	6*3*3
4	Male	60	pTaNxMx	3.5*2.5*0.8
5	Male	70	pT1NxMx	7.5*3*1.5
6	Male	81	pTaNxMx	3*2*0.5
7	Male	68	pTaNxMx	3*2.5*1
8	Male	79	pTaNxMx	3.5*3*1
9	Male	76	pT2aNxMx	4.5*3*2
10	Male	41	pT3aNxMx	4*3*0.8
11	Male	67	pT3bN2Mx	4*2.5*2
12	Male	69	pT4N0Mx	4*3*1
13	Male	64	pT4bN2Mx	2*1*1
14	Male	51	pT3bN0Mx	3*2*1
15	Male	52	pT4aN1Mx	3*2.5*1
16	Male	65	pT2bN0Mx	4*3*1

Table S2. Clinicopathological characteristics of the HBlaU079Su01 cohort.

	**Variables**	**Total (n=63)**	**USP52 expression High (n=32)**	**USP52 expression Low (n=31)**	***p*-value**	**Statistics method**
Gender (%)	Female	10 (15.87)	7 (21.88)	3 (9.68)	0.3020	Fisher's exact
	Male	53 (84.13)	25 (78.12)	28 (90.32)		
Age (year) (%)	≤ 65	21 (33.87)	11 (34.38)	10 (32.26)	0.7884	Chi-square
	> 65	41 (66.13)	20 (62.50)	21 (67.74)		
Subtype (%)	MIBC	42 (72.41)	30 (93.75)	12 (38.71)	**<0.0001**	Chi-square
	NMIBC	16 (27.59)	2 (6.25)	14 (45.16)		
AJCC stage (%)	< Stage II	11 (20.37)	2 (6.25)	9 (29.03)	**0.0041**	Fisher's exact
	≥ Stage II	43 (79.63)	30 (93.75)	13 (41.94)		
T (%)	< T2	16 (27.59)	2 (6.25)	14 (45.16)	**0.0001**	Chi-square
	≥ T2	42 (72.41)	29 (90.63)	13 (41.94)		
N (%)	N ≥ 1	8 (15.09)	5 (15.63)	3 (9.68)	0.7153	Fisher's exact
	N0	45 (84.91)	24 (75.00)	21 (67.74)		
Tumor Size (%)	≤ 3 cm	18 (31.58)	8 (25.00)	10 (32.26)	0.5093	Chi-square
	> 3 cm	39 (68.42)	21 (65.63)	18 (58.06)		

USP52 expression group: The median of the USP52 average optical density was cut-off value;

Tumor size: The longest diameter, cm;

Statistical significance: Determined by two-tailed Chi-square or two-tailed Fisher's exact test. No adjustments were made for multiple comparisons.

Table S3. The siRNA sequences used in this study.

siRNA	sense (5'-3')	antisense (5'-3')
NC	UUCUCCGAACGUGUCACGUTT	ACGUGACACGUUCGGAGAATT
siUSP52.1	CUGUCUACCUGUUCCAUAUTT	AUAUGGAACAGGUAGACAGTT
siUSP52.2	GCAUGAUCCAGGUGCUCUATT	UAGAGCACCUGGAUCAUGCTT
siUSP52.3	GAGUUACGCAGUGAUGGUATT	UACCAUCACUGCGUAACUCTT

Table S4. The shRNA sequences used in this study.

shRNA	sequences (5'-3')
shNC	TTCTCCGAACGTGTCACGT
shUSP52	GCTTCCTTTCTCCATTCGCAT

Table S5. Primers used for qRT-PCR assays.

Gene	Forward primer (5'-3')	Reverse primer (5'-3')
USP52	GTGGGTGTACCTGTTTCCGTC	GCTCTGGATCTGCCGAATATCA
xCT	TGGTCAGAAAGCCTGTTGTGT	ACCGTCCAGATGGTCAGAGA
GAPDH	GGAGCGAGATCCCTCCAAAAT	GGCTGTTGTCATACTTCTCATGG
ACSL4	CATCCCTGGAGCAGATACTCT	TCACTTAGGATTTCCCTGGTCC
ACSL5	TGGACATCAGGTCACGTTGG	GGCTTTGACTTGGTTTTGGCA
AHCYL1	CGCTCGATCTCACAGTCCTC	TGAACACCAGTCACGCTCTC
CHAC1	GAACCCTGGTTACCTGGGC	CGCAGCAAGTATTCAAGGTTGT
CP	GCCACAAGGCCCTACTCAAT	CAGCCAGATTTGGTGTCTTCATTT
DNMT3B	CCAACAACACGCAACCAGAG	CTGCCACAAGACAAACAGCC
FTH1	GCTCTACGCCTCCTACGTTT	GTGGCCAGTTTGTGCAGTTC
FTL	AGCCAGCTGAAGATGAGTGG	TCCAAAAGGGCCTGGTTCAG
GGCT	CACCCTTGCAAGCACAAGAC	AGGTGCCATGACCTGATTGG
GGT1	GTACCACCGCATCGTAGAGG	AGAGGTTGATGGTGCTGGTG
HMOX1	ACTCCCTGGAGATGACTCCC	TCTTGCACTTTGTTGCTGGC
LACC1	CTGCCAGCACTGAGAGGAAA	TGCCTCTGCTGATTCCCTTG
MTAP	CACTCGGGACTCACTTGCC	TGTTCGCCTGGTAGTTGACC
ODC1	TGATGCCCGCTGTGTTTTTG	AACTGCAAGCGTGAAAGCTG
SAT1	CTCCGGAAGGACACAGCATT	ACCTCATTGCAACCTGGCTT
SLC11A2	GTGGTTACTGGGCTGCATCT	AGACTTCAACCACCTGCTCG
SLC39A8	TTTGAAGGGGCCCAAACTGT	AGAGACAAGGTGCAGGAAGC
TST	TCTGGTGGATGTTCCGTGTG	CCGCCCTTGAGACCTTGAAT

qRT-PCR: Quantitative reverse transcription PCR.

Table S6. Details of antibodies used in this study.

Antibody	Catalog No.	Source	Dilution or amount
Flag-tag	F1804	Sigma	IP/1 μg; WB/1:1000
HA-tag	AE105	ABclonal	WB/1:1000
Myc-tag	AE010	ABclonal	IP/1 μg; WB/1:1000; IF/1:200
USP52	16427-1-AP	Proteintech	IP/2 μg; WB/1:1000; IHC/1:200
xCT	26864-1-AP	Proteintech	WB/1:1000; IHC/1:200
xCT	ab175186	Abcam	IP/2 μg; WB/1:1000
xCT	HA600098	HuaBio	IF/1:100
GAPDH	60004-1-Ig	Proteintech	WB/1:10000
ACSL4	ab155282	Abcam	WB/1:1000
GPX4	ab125066	Abcam	WB/1:1000
Ki67	ab16667	Abcam	IHC/1:200
Ubiquitin	ab7254	Abcam	WB/1:1000
4-HNE	ab48506	Abcam	IHC/1:200
BAX	2772S	Cell Signaling	WB/1:1000
Bcl-2	2870S	Cell Signaling	WB/1:1000
p62	ab56416	Abcam	WB/1:1000
LC3B	2775	Cell Signaling	WB/1:1000
GSDMD	39754S	Cell Signaling	WB/1:1000
Caspase 1	24232S	Cell Signaling	WB/1:1000

IP: Immunoprecipitation;

WB: Western blot;

IF: Immunofluorescence;

IHC: Immunohistochemistry.

Table S7. Details of compounds used in the study.

Reagents	Catalog No.	Source
Erastin	S7242	Selleck
TBH	458139	Sigma
Ferrostatin-1	HY-100579	MedChemExpress
Z-VAD-FMK	HY-16658B	MedChemExpress
3-Methyladenine	HY-19312	MedChemExpress
MG132	HY-13259	MedChemExpress
CHX	HY-12320	MedChemExpress
IKE	S8877	Selleck
Disulfiram	S1680	Selleck
